# Insertions/Deletions-Associated Nucleotide Polymorphism in *Arabidopsis thaliana*

**DOI:** 10.3389/fpls.2016.01792

**Published:** 2016-11-30

**Authors:** Changjiang Guo, Jianchang Du, Long Wang, Sihai Yang, Rodney Mauricio, Dacheng Tian, Tingting Gu

**Affiliations:** ^1^State Key Laboratory of Pharmaceutical Biotechnology, School of Life Sciences, Nanjing UniversityNanjing, China; ^2^Provincial Key Laboratory of Agrobiology, Institute of Biotechnology, Jiangsu Academy of Agricultural SciencesNanjing, China; ^3^Department of Genetics, University of GeorgiaAthens, GA, USA; ^4^State Key Laboratory of Plant Genetics and Germplasm Enhancement and College of Horticulture, Nanjing Agricultural UniversityNanjing, China

**Keywords:** structural variation, insertion, deletion, nucleotide polymorphism, nucleotide dimorphism

## Abstract

Although high levels of within-species variation are commonly observed, a general mechanism for the origin of such variation is still lacking. Insertions and deletions (indels) are a widespread feature of genomes and we hypothesize that there might be an association between indels and patterns of nucleotide polymorphism. Here, we investigate flanking sequences around 18 indels (>100 bp) among a large number of accessions of the plant, *Arabidopsis thaliana*. We found two distinct haplotypes, i.e., a nucleotide dimorphism, present around each of these indels and dimorphic haplotypes always corresponded to the indel-present/-absent patterns. In addition, the peaks of nucleotide diversity between the two divergent alleles were closely associated with these indels. Thus, there exists a close association between indels and dimorphisms. Further analysis suggests that indel-associated substitutions could be an important component of genetic variation shaping nucleotide polymorphism in *Arabidopsis*. Finally, we suggest a mechanism by which indels might generate these highly divergent haplotypes. This study provides evidence that nucleotide dimorphisms, which are frequently regarded as evidence of frequency-dependent selection, could be explained simply by structural variation in the genome.

## Introduction

One of the fundamental discoveries in evolutionary genetics is that the distribution of nucleotide substitutions in the genomes of living organisms is not random. This non-random distribution can be seen in a variety of ways: mutational hot-spots (Iacobuzio-Donahue et al., 2004; Galtier et al., 2006), high levels of within-species diversity (Hughes and Nei, 1988; Noël et al., 1999; Bergelson et al., 2001), and structured polymorphisms (Du et al., 2008).

There exist several well-known examples of highly polymorphic genomic regions, including the human major histocompatibility complex (*MHC*) (Hughes and Nei, 1988) and plant disease resistance genes (*R*-gene) (Noël et al., 1999; Stahl et al., 1999; Bergelson et al., 2001). The average nucleotide diversity (π) at the *MHC* loci is about 10 times higher than that at the other nuclear loci (Hughes and Nei, 1992), and the level of polymorphism in *R*-gene in *Arabidopsis thaliana* is 9–42 times higher than that of the other regions (Noël et al., 1999; Nordborg et al., 2005). The extremely high levels of polymorphism within species are sometimes even higher than the divergence between closely related species (Stahl et al., 1999; Tian et al., 2002). In general, lower levels of nucleotide polymorphism are expected within species than between species, because reproductive isolation between species should lead to the accumulation of high levels of between-species genetic divergence. Within species, many evolutionary forces, including gene conversion, can deplete nucleotide polymorphism.

A relatively recently described form of nucleotide polymorphism is the dimorphism, found when nucleotide variation around some loci in a population sample can be clearly partitioned into two distinct sets of haplotypes. Such dimorphisms have been described in *Arabidopsis* (Hanfstingl et al., 1994; Stahl et al., 1999; Aguadé, 2001; Tian et al., 2002) and the fruitfly (*Drosophila melanogaster*) (Teeter et al., 2000; Wang et al., 2002). The maintenance of dimorphic regions in the genome is also surprising as we would expect them to be quickly eroded by recombination.

Efforts have been made to understand the origin and maintenance of highly divergent haplotypes. Overdominance is one of the most popular mechanisms to explain them, and is, for example, suggested to be responsible for the extreme diversification at the *MHC* loci (Hughes and Nei, 1988; Li, 1997; Hughes and Yeager, 1998). Within highly selfing species, such as *A. thaliana*, however, overdominance is likely to be a much less effective mechanism due to the lack of heterozygosity (Bergelson et al., 2001). Balancing selection has been proposed to be responsible for high levels of genetic variation around some dimorphic loci in *A. thaliana* (Stahl et al., 1999; Bergelson et al., 2001; Tian et al., 2002; Shen et al., 2006). But the relative long time required for balancing selection cannot explain the commonly observed dimorphisms in the whole genome (Du et al., 2008). In spite of an extensive number of studies (e.g., Kawabe et al., 1997; Kuittinen and Aguadé, 2000; Yoshida et al., 2003), no general mechanism has been suggested to explain how such distinct sets of haplotypes with extreme polymorphic variation arise and are maintained.

We propose a novel mechanism based upon recent observations that insertions or deletions (indels) locally suppress crossovers (Hammarlund et al., 2005; Ziolkowski et al., 2015), and increase mutation rate directly (Tian et al., 2008; Conrad et al., 2010a,b; De and Babu, 2010; Hollister et al., 2010) or indirectly (McDonald et al., 2011). Therefore, in regions adjacent to the insertion/deletion junction, we might expect reduced recombination rate, increased mutation rate and increased polymorphism between the insertion-present and -absent haplotypes. Thus, mutations linked to the indel site could occur and accumulate more rapidly over time between the two haplotypes than within haplotypes. An indel could act as a regional “genetic isolator” (due to suppressed recombination) or local “mutator” (due to increased mutation) between two haplotypes. This would lead to a higher divergence in the regions close to indels between them, a signature of “indel-associated polymorphism” or a pattern of dimorphism that should be primarily affected by mutation and neutral drift.

Our “indel-associated polymorphism” model has several predictions. First, there should be a close association between indels and dimorphisms: nucleotide dimorphisms should be found near indels, and conversely, indels should be identified close to dimorphisms. Second, the effect of increased mutation and suppressed recombination around indels should lead to an indel-centered distribution of divergence between haplotypes. Third, the association between indels and dimorphism should be specific to indels. The indels with different features, such as locations, sizes and GC content, should have a different effect on the performance of the associated polymorphism.

We tested these predictions by examining genomic data collected from *Arabidopsis thaliana. Arabidopsis* is particularly suitable for such study because it is highly self-fertilizing (Abbott and Gomes, 1989). Thus, its low rate of effective recombination helps preserve the signature of indel-associated nucleotide polymorphism. We sequenced and investigated the flanking sequences around 18 indels (>100 bp) and four long intergenic regions. Dimorphisms are present around all these indels and throughout the intergenic sequences, and indels are always associated with previously identified dimorphic loci. Furthermore, analysis of other large-scale datasets, the Nordborg dataset (1214 loci sequenced in 96 *Arabidopsis thaliana* accessions Nordborg et al., 2005; and the 81 whole genome sequences of *Arabidopsis thaliana* produced by 1001 Genome Project Cao et al., 2011; Alonso-Blanco et al., 2016), supports the predictions. Our results demonstrate a close association between indels and dimorphism and suggest a mechanism for the origin and maintenance of highly divergent alleles.

## Materials and methods

### Selection of indel loci for evidence of nucleotide dimorphism

The 746 large insertion-deletion polymorphisms (>100 bp) between the Columbia (Col-0) and Landsberg *erecta* (L*er*) accessions originally identified by Jander et al. (2002) (http://www.arabidopsis.org/Cereon) were used for the selection of indel loci. These indels are assumed to be insertions in Col-0 relative to L*er* (or deletions in L*er* relative to Col-0). We screened the indels manually on the published Col-0 genome (version 9) and excluded 179 loci, as they either overlapped with other indels or were less than 100 bp in length. Of the remaining indels, 388 were between 100 and 2 kb in length and 179 indels were >2 kb (Supplementary Table S1).

We searched for the 388 smaller indel sequences in the *Arabidopsis thaliana* genome using the Basic Local Alignment Search Tool (BLAST) (http://www.ncbi.nlm.nih.gov) (Altschul et al., 1990). In an attempt to avoid repetitive sequence or transposable elements that might be difficult to sequence, we discarded any insertion with sequence hits >1 in a BLAST search. For the remaining 174 indels, we attempted to search the incomplete L*er* genome sequences by using ~1 kb up- and down-stream flanking sequences around the indel in the Col-0 genome. In this round of screening, 38 indel loci were excluded due to unavailable sequences in L*er*. Based on previous study (Du et al., 2008), a dimorphic locus can be clearly identified if there was a nucleotide diversity of 0.01 to 0.05 (3 or more SNPs per 500 bp sequence) between two haplotypes. Therefore, 13 indel loci, the nucleotide diversity of which ranged from 0.01 to 0.05 between L*er* and Col-0, were randomly selected from the remaining 136 indels, and genotyped for indels in at least 16 *Arabidopsis* accessions (Supplementary Table S2). In these cases, we sequenced a ~1 kb flanking region spanning the breakpoints of the indels.

Of the 179 larger indels (>2 kb), we sampled five indels which contained gene(s) (but not transposons or retrotransposons), and genotyped 40–44 worldwide accessions of *A. thaliana* for each locus. Two loci were sequenced because of their intermediate frequencies of indels among populations (one with 16/42 of insertion and another with 18/43 of deletion haplotype). Meanwhile, three indels with disease resistance (*R*) genes were sampled because these genes were confirmed as ancient presence/absence polymorphisms (Shen et al., 2006). The three sequenced *R*-gene loci are named *R*-Gene Dimorphism loci (RGD1–3) and the other 15 non-*R* gene loci are named Non-*R*-Gene Dimorphism loci (NRD1–15, Supplementary Figure S1). Please see Supplementary Data Sheet 1 for the details of the location information and sequence alignment of selected loci.

### Selection of long intergenic loci for sequencing

To investigate the indel and polymorphic patterns in regions with little selection, we examined polymorphism in long intergenic regions thought to be evolving neutrally. We identified 565 long intergenic regions in the Col genome (TAIR9) by two criteria: long intergenic sequences (>8 kb) and <15% of repeat sequences. Four long intergenic loci (LI1–4) were randomly sampled for further sequencing in 12–16 *Arabidopsis* accessions. Locus 1 is located 9736779–9744965 bp of chromosome 3 (8.2 kb long), locus 2 at position 6201213–6209722 bp (8.5 kb long) of chromosome 4, locus 3 at position 22759961–22771327 bp (11.4 kb long) of chromosome 1, locus 4 at position at 18763958–18772537 bp (8.6 kb long) of chromosome 5.

### Selection of long indels from the 1001 genome project to analyze the surrounding SNPs that link to the indels

The genomic sequences of 81 *Arabidopsis thaliana* accessions (Cao et al., 2011) (data from 1001 Genome Project) were searched for large indels with relatively high quality sequence data. To be consistent with the criteria of indel picking described in the first section of Methods, the indels satisfying the following criteria were picked from the 1001 Genome Project: (1) the size of the indels are >100 bp; (2) the deletion junction of the indels could be clearly identified; (3) in the 2 kb upstream and downstream flanking sequences, no indels are longer than 10 bp; (4) the insertion sequence itself contains no more than 1% ambiguous nucleotides (ambiguous nucleotides are those denoted as “Z”-zero coverage or “N”-no call possible in the original sequences downloaded from the 1001 Genome Project datacenter); (5) the 2 kb flanking sequences contain no more than 5% ambiguous nucleotides. Finally, 82 indel loci met those criteria were picked for further analysis.

### Genotyping and sequencing

In this study, we used a total of 65 accessions of *A. thaliana* from worldwide samples (Supplementary Table S2). PCR amplification allowed us to determine the presence/absence of the selected indels and to amplify the flanking regions around each of the selected indel loci (RGD and NRD loci) for sequencing. For each of the large indel loci (locus RGD1–3 and NRD1–2), a three-primer PCR was used (two primers are designed in the 3′ and 5′-flanking region of the break point, and one in the insertion sequence) to give alternative products for the indel-present or -absent genotype. For the locus NRD3–15 (indel sizes <1.5 kb), a two-primer PCR was used for genotyping. Genotyping revealed an average of 33.7% indel frequency (ranging from 16.7 to 57.1%) among these accessions (Supplementary Table S2). Based on the results of genotyping and of simulation for the proper accession number required as samples (Supplementary Figure S2, Results for details), 8–20 accessions were randomly selected from both of the indel-present or -absent genotypes for sequencing. The sequenced regions were located 0.5–8 kb away from the deletion junction for each locus. All sequencing reactions were run on an ABI 3100-Avant automated sequencer.

To rule out the possibility of PCR contamination in our sequencing, the Perlegen dataset (Clark et al., 2007) was used to check the consistency with our sequencing results. This dataset did not have information for large indels. Therefore, we first did PCR genotyping to determine the indels at the 15 single-indel loci for 10 accessions, Bur-0, C24, Cvi-0, Got-7, Lov-5, Rrs-7, Rrs-10, Tamm-2, Ts-1, and Tsu-1 (randomly sampled from the 20 accessions in the Perlegen data). The PCR results confirmed that all loci have the same indels as we obtained. In addition, in all the eight loci having informative SNPs (single nucleotide polymorphism) in the Perlegen data, the polymorphic patterns were stratified into two groups consistent with the indel present/absent pattern (Supplementary Figure S3). Thus, the indel-associated dimorphic SNPs observed in our sequenced 18 loci are unlikely the result of PCR contamination.

### Analysis of polymorphisms

A dimorphism is identified based on the extended dimorphic sites. The minor haplotype is denoted as haplotype x with n_1_ accessions, and the major haplotype as y with n_2_ accessions in a dimorphic locus. Thus, at a dimorphic locus, the nucleotide diversity (π_t_ Nei, 1987), can be divided into three parts: the contribution by mutations within haplotypes (π_*h*_), the fixed substitutions (π_*fixedxy*_) and the non-fixed substitutions (π_*non*−*fixedxy*_) between haplotypes:

(1) $$\begin{array}{l} \pi_{t}=\frac{n_{1}(n_{1}-1)}{n(n-1)}\times \pi_{x}+\frac{n_{2}(n_{2}-1)}{n(n-1)}\times \pi_{y}\\ \;+\frac{2n_{1}n_{2}}{n(n-1)}\times D_{xy} \end{array}$$

(2) $$\begin{array}{rc} = & \pi_{h_{1}}+\pi_{h_{2}}+\left(\pi_{fixedxy}+\pi_{non-fixedxy}\right) \end{array}$$

(3) $$\begin{array}{rc} = & \pi_{h}+\pi_{fixedxy}+\pi_{non-fixedxy} \end{array}$$

Where $\pi_{fixedxy}=\frac{2n_{1}n_{2}}{n(n-1)}\times d_{xy}$, and *D*_*xy*_ is the divergence between haplotypes. Not the same as *D*_*xy*_, the d_xy_ is the fixed nucleotide divergence between haplogroups, equal to the total number of fixed substitutions divided by the total length of corresponding sequence (Supplementary Figure S4). The weighted *d*_*xy*_ – π_*fixedxy*_ is the component of nucleotide variation contributed by fixed substitutions. Therefore, the ratio of π_*fixedxy*_/π_*t*_ reflects the relative contribution of the fixed nucleotide dimorphic-sites to the total nucleotide variation.

### Simulation analysis

To detect whether indel-linked SNPs are random or not, we performed coalescent simulations of the probability of a 600 bp locus containing a certain numbers of linked SNPs. The simulation is based on a neutral model with constant population size, no recombination, panmixis, and infinite sites. We repeated the simulation 10000 times, using software developed by Hudson (2002). The mutation rate (θ) is 4 [=S/1215/ $\overset{n-1}{\underset{j=1}{\sum }}\frac{1}{j}$ /580^*^600]; S is the sum of SNPs and indels obtained from the Nordborg dataset (Nordborg et al., 2005), the sequence length is set as 600 bp, the same length as the junction regions in the sequenced 18 indel loci]. If the probability of a locus having more than 3 mutually linked SNPs is <0.05 in selected accessions (=n), the dimorphic loci would be not random.

## Results

### Association of indels with highly divergent dimorphic alleles

We sequenced and investigated the pattern of nucleotide polymorphism of flanking sequences around 18 large indels (>100 bp). Among them, 15 Non-*R* gene Dimorphism loci (NRD1–15) were sampled from an indel database of *Arabidopsis thaliana* (Jander et al., 2002) by a set of criteria (e.g., >100 bp and non-repetitive, Materials and Methods for details), and the other three *Resistance* Gene (*R*-gene) Dimorphism loci (RGD1–3) were picked from those indels containing *R*-gene (Shen et al., 2006). In addition, two other large indels containing *R*-gene *rpm*1 and *rps*5 (Stahl et al., 1999; Tian et al., 2002) were included in the analysis also. Because of their size and central importance to the selection of the loci studied, we refer to these indels as the “major” indels corresponding to each locus. We first confirmed that each of the 18 loci contained an indel polymorphism by amplifying the region across the deletion junction in 21–59 *Arabidopsis* accessions (Supplementary Table S2). Then 8–20 accessions, randomly sampled from genotyped ones to represent both insertion-present and -absent haplotypes, were sequenced in the junction regions (defined as the ~600 bp sequences surrounding the deletion junction).

Fifteen of the 18 loci contained a single indel polymorphism, and three (locus NRD13–15) contained two different indels (different indel size and position, Table 1). Among the 18 loci, the indel sizes range from 4243 to 6266 bp in three *R*-gene insertions (locus RGD1–3), from 4524 to 5584 bp in two insertions containing other functional genes (locus NRD1 and NRD2), 1397 bp in one insertion containing a pseudogene (locus NRD11), and from 101 to 1076 bp in 12 loci with insertions in non-coding sequences (NRD3–10, 12–15).

**Table 1:** **Statistics for five present/absent ***R***-genes and 15 other indel loci in the junction region**.

**Type**	**Locus**	**Indel size (bp)**	**Position to DJ**	**Sequenced accessions**	**Length (bp)**	**Fixed sites**		**Nucleotide diversity**				**Tajima's D**
						**S_N_**	**indel**	**π_t_**	**π_fixed_**	**D_xy_**	**d_xy_**	
RGD	1	4243	5′-4.7 kb	21	643	0	0	0.0051	0	**/**	0	−0.98
			5′-0.3 kb	21	1088	0	0	0.0091	0	**/**	0	1.49
			JR	21	600	37	5	**0.0366**	**0.0329**	**0.0713**	**0.0628**	2.86^**^
			3′-0.3 kb	21	1143	19	5	0.0128	0.009	**/**	0.0172	1.17
			3′-4.5 kb	21	597	0	0	0.0036	0	**/**	0	−0.84
	2	5057	5′-5.7 kb	20	601	0	0	0.0144	0	**/**	0	−0.72
			5′-2.2 kb	21	605	0	0	0.0073	0	**/**	0	1.55
			5′-JR	21	618	49	12	**0.0517**	**0.0468**	**0.0952**	**0.0894**	3.28^**^
			3′-1.4 kb	21	532	0	0	0.001	0	**/**	0	1.57
			3′-4.3 kb	21	719	25	2	0.0202	0.0192	**/**	0.0368	2.51^**^
			3′-8.0 kb	21	698	0	0	0.0058	0	**/**	0	−0.33
	3	6266	5′-4.9 kb	21	553	0	0	0.0046	0	**/**	0	0.43
			5′-2.1 kb	21	610	8	2	0.028	0.011	**/**	0.0223	1.49
			5′-0.3 kb	21	1161	9	3	0.0236	0.0144	**/**	0.0297	1.6
			JR	21	600	15	3	**0.0226**	**0.0126**	**0.0387**	**0.027**	2.21^*^
			3′-3.0 kb	21	568	0	0	0.0049	0	**/**	0	−1.22
	*Rpm1*	3764	JR	28	600	37	10	**0.0396**	**0.0373**	**0.0799**	**0.073**	3.07^**^
	*Rps5*	3990	JR	22	600	24	3	**0.026**	**0.0219**	**0.0497**	**0.0423**	2.62^**^
NRD	1	4524	JR	21	600	4	0	**0.0092**	**0.0035**	**0.0115**	**0.0067**	**0.84**
	2	5584	JR	17	600	14	3	**0.0197**	**0.0109**	**0.0332**	**0.0233**	**1.77**
	3	1076	JR	17	600	27	1	**0.0287**	**0.0261**	**0.051**	**0.0417**	2.51^**^
	4	807	JR	9	600	31	11	**0.0268**	**0.0247**	**0.031**	**0.0417**	2.31^**^
	5	404	JR	9	600	11	5	**0.0123**	**0.0038**	**0.0192**	**0.0069**	**1.03**
	6	101	JR	9	600	30	4	**0.0067**	**0.0056**	**0.0118**	**0.01**	**1.71**
	7	578	JR	8	600	5	1	**0.0078**	**0**	**0.0101**	**0**	**0.47**
	8	121	JR	9	600	3	0	**0.0138**	**0.0009**	**0.0139**	**0.0017**	−1.80^*^
	9	135	JR	9	600	34	6	**0.019**	**0.014**	**0.032**	**0.0252**	**1.65**
	10	1001	JR	9	600	2	1	**0.0042**	**0.0009**	**0.0052**	**0.0017**	−**1.13**
	11	1397	JR	9	600	9	5	**0.0057**	**0.0019**	**0.0083**	**0.0034**	**0.6**
	12	304	JR	9	600	8	0	**0.0128**	**0.0047**	**0.0166**	**0.0084**	−**0.04**
	13	238/501	JR	9	600	/	/	**0.0133**	**/**	**/**	**/**	**0.39**
	14	345/340	JR	8	600	/	/	**0.0089**	**/**	**/**	**/**	−**1.18**
	15	205/335	JR	9	600	/	/	**0.0058**	**/**	**/**	**/**	−**0.89**

*More information on the loci is in Supplementary Table S2 and Supplementary Figure S1. DJ, JR, and S_N_ represent deletion junction, junction region and the nucleotide substitution sites, respectively*.

*, P < 0.05;

**, *P < 0.01. The values in bold represent the nucleotide diversity in the JR. The flanking sequences of Rpm1 and Rps5 were obtained from Genbank (Stahl et al., 1999; Tian et al., 2002)*.

**Figure 1:**
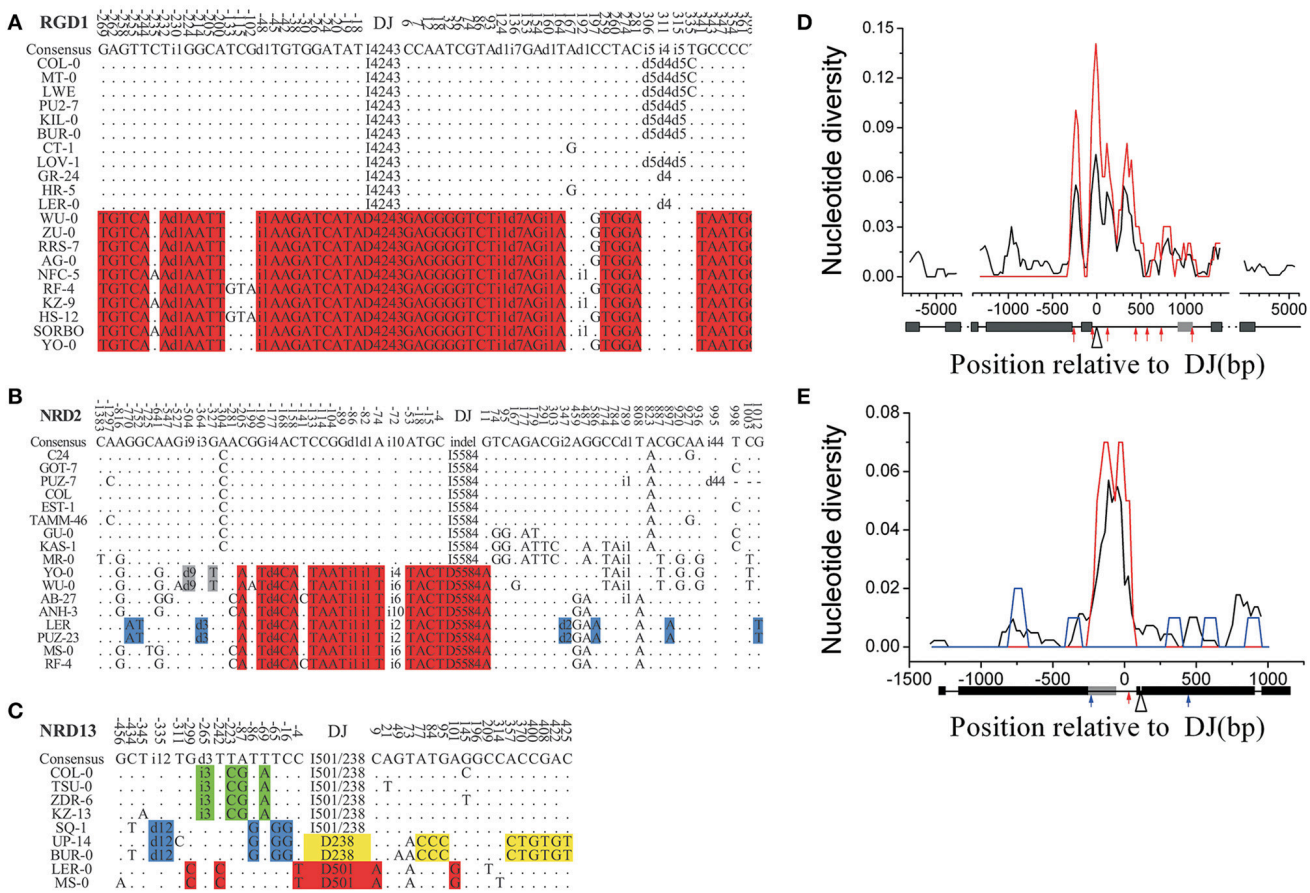
**Polymorphism in locus RGD1, NRD2, and NRD13 (A–C)** and sliding window analysis of the nucleotide diversity (black line) and the dimorphic diversity (d_xy_, red line) in RGD1 **(D)** and NRD2 **(E)**. **(A–E)**: The I/i4243 or D/d4243 designations represent either a 4243-bp insertion or deletion. The different colored sites at each locus represent different patterns of nucleotide dimorphisms linked to an indel. Fixed mutations in a haplogroup are marked with red or yellow background, while the mutations in compound dimorphic patterns are green- or blue-colored. **(D–E)**: The number (bp) on the horizontal axis represents the relative position to the deletion junction (DJ) at each locus. Selected indels are positioned at 0. The red line represents the corresponding d_xy_ contributed by the SNPs linked to the selected indel (the open triangle). Black and gray boxes represent coding regions and 5′- or 3′-UTR, respectively. Red arrows marked the major indels with the same dimorphic pattern, while the minor indels were marked as blue arrows.

We observed a clear nucleotide dimorphic pattern around all the 15 single-indel loci (Table 1, Figures 1A–C, Supplementary Table S3 and Supplementary Figure S5). At these loci, 3–88 fixed mutations per locus were identified between the insertion-present and -absent haplotypes, including 340 substitutions and 70 indels in total, in the sequenced junction regions (Table 1). All indel variations fixed within the haplotypes were masked prior to calculating the divergence between the two haplotypes. After excluding those fixed indels, the fixed nucleotide diversity (d_xy_) ranges from 0 to 0.0894 (0.0241 on average) in the junction regions. The percentage of the π_fixedxy_ to the total nucleotide diversity at junction regions (π_t_ = 0.0185 on average) ranges from 0 to 92.2% (54.5% on average), indicating that the fixed substitutions are important components of genetic variation.

A higher Tajima's *D* value, which is suggested to be often present at dimorphic loci (Hanfstingl et al., 1994), was also observed in the junction region. The average Tajima's *D* for the junction regions of the 15 loci was 1.2 (absolute value), significantly higher than the value for genome-wide samples (−0.8 in 876 loci from Nordborg Dataset (Nordborg et al., 2005), *P* < 0.0001, paired *t*-test). Moreover, the average nucleotide divergence between the insertion-present and -absent haplotypes (D_xy_) is significantly larger than the average nucleotide diversity among the sequencing alleles (π_t_, Table 1, *P* < 0.05, paired *t*-test) in the junction regions, arguing that there is a specific mechanism maintaining the high divergence between the two haplotypes.

**Figure 2:**
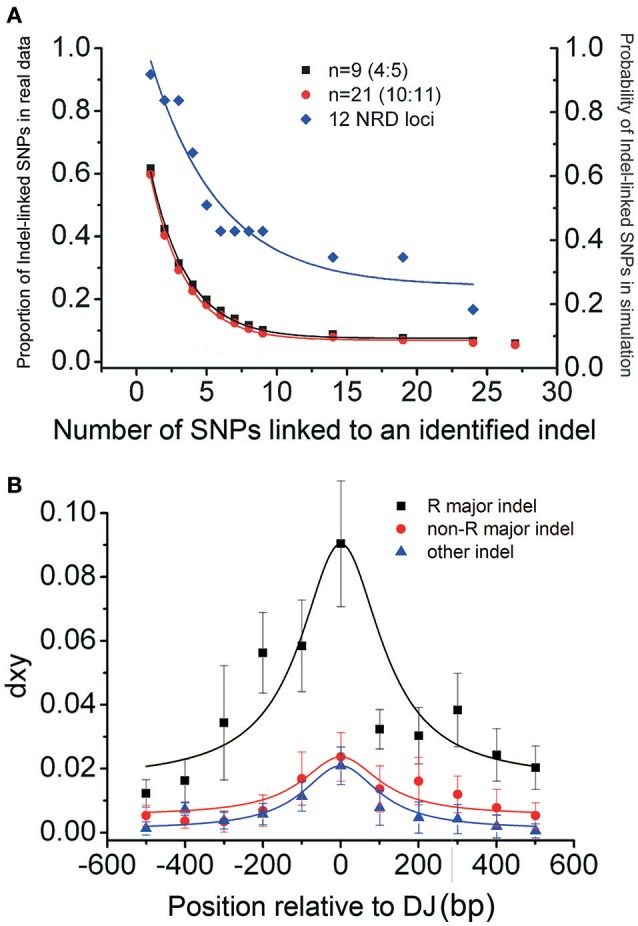
**Distribution of nucleotide substitutions around indels in the RGD and NRD loci shows a close association between indels and nucleotide dimorphisms. (A)** The probability of a 600-bp locus containing certain numbers of SNPs linked to a previously identified indel expected by the neutral model (the bottom two lines) and proportion of linked SNPs observed in the NRD1–12 loci (the top line). Black (or red) lines are from the simulations assuming that there are nine (or 21) accessions, and the indel frequency is 4:5 (or 10:11). The observed dimorphic substitutions in the 12 NRD junction regions (blue line) are overall significantly higher than the simulated results (Supplementary Figure S2 legend for more details). **(B)** Relationship between d_xy_ and distance from major indels at five RGD loci (black-filled rectangle), at NRD1–12 (black-filled circle), and from the other 25 indels at the loci above (blue-filled triangle). Selected indels are positioned at 0. The standard error of mean (SEM) was denoted as error bar for each point.

To confirm that those indel-linked SNPs are not random, simulation analysis was performed based on Hudson's model (Hudson, 2002). The mutation rate (θ) used for simulation was estimated from the genome-wide sequencing data in 96 accessions in the Nordborg Dataset (Nordborg et al., 2005). The simulation showed that the number of fixed SNPs observed around major indels was significantly larger than that expected by a neutral model (Figure 2A, one-phase exponential decay). For example, eight out of the sequenced 12 single-indel loci (NRD1–12) contained more than 3 dimorphic sites corresponding to the major indels, significantly higher than random expectation (*P* < 0.05, chi-square test). Meanwhile, compared to 21 accessions, nine accessions assayed per indel locus did not lose much power to declare the dimorphic patterns (Figure 2A and Supplementary Figure S2). Thus, the close association between indels and nucleotide dimorphisms, originally observed in a limited number of accessions in the 18 indel loci, does not seem random, but indicates an indel-associated mechanism influencing the distribution of the nucleotide polymorphic patterns around.

Our indel-associated polymorphism model also predicts that the level of divergence between the two haplotypes should reach a maximum around the indel. This expected indel-centered distribution of fixed mutations was observed by nucleotide diversity and d_xy_ sliding window analyses at the 15 single-indel loci (Figures 1D–E and Supplementary Figure S6). The analysis shows that d_xy_ is highest immediately surrounding the deletion junction of each sampled indel. The highest peak is located within 300 bp of the deletion junction in 10 out of 15 loci and within 700 bp in all loci except for indel-locus NRD6, which contained a highest peak around 800 bp away from the deletion junction. The average divergence at these peaks is 0.082 (ranging from 0.020 to 0.206), reflecting an extreme variation in these loci. Notably, the indel-centered d_xy_ distribution, observed around the major indels (red arrows), is also present around minor indels (blue arrows) (Figures 1D–E: e.g., at 500 bp of RGD1 and 750 bp of NRD5).

This indel-centered distribution of d_xy_ was further confirmed by the correlation analysis (Figure 2B). There exists a clear negative correlation between d_xy_ and the distance to their corresponding deletion junctions at RGD loci (black curve; *R*^2^ = 0.90; *P* < 0.05) and the other NRD loci (red curve; *R*^2^ = 0.90, *P* < 0.01). d_xy_ reaches its maximum within 100 bp to the deletion junction and decays very quickly. The *R* loci show an average d_xy_ of 0.1 around the deletion junction, higher than that of non-*R* gene loci, indicating that balancing selection is also functioning in those *R*-gene loci (discussed below). While in the NRD loci, the DNA polymorphism around the major indel also gives an indel-centered d_xy_ distribution, but the diversity level is much lower and fading more quickly. Interestingly, the indel-centered d_xy_ is also seen around the small indels (other than major indels) in those loci, and the extension of the dimorphisms is even shorter (blue curve). This observation confirms that this indel-centered d_xy_ distribution is a general pattern to indels, and that longer indel may have a major impact due to its more effective recombination suppression effect. The rapid decay of d_xy_ indicates that the average extension of a dimorphism in non-*R* loci is about 1 kb, and that the average extension of a dimorphism in *R* loci is no more than 10 kb (Figure 2B), consistent to the observations of previously reported insertion/deletion *R*-genes (Stahl et al., 1999; Aguadé, 2001; Tian et al., 2002).

Interestingly, some loci were found to contain more than one type of dimorphism, the compound dimorphisms. Compound dimorphic patterns were observed at loci with two different indels (locus NRD13–15). At locus NRD13, for example, there are two deletions, a 238 bp (D238) and a 501 bp deletion (D501) relative to Col-0 at the deletion junction. Nine (yellow-colored) and five fixed mutations (red-colored) were identified corresponding to these two types of deletions, respectively (Figures 1A–C). Also, additional dimorphisms, corresponding to different indel patterns, could be identified at this locus. For example, the other two dimorphic patterns with three fixed sites each (green- and blue-colored) could be identified corresponding to the 3 bp and 12 bp deletion, respectively. The compound dimorphisms, which correspond to different types of indels, provide further evidence for the specific association between indel and corresponding nucleotide substitutions.

### Association between indel polymorphisms and previously identified dimorphic loci

To further confirm the close association between indels and nucleotide dimorphism, we examined the flanking sequences of some known nucleotide dimorphism loci to see whether there were linked indels around. The Nordborg dataset (Nordborg et al., 2005) contains the aligned sequences of 96 *A. thaliana* accessions for each of 1214 loci. First, we excluded 84 loci due to insufficient accession sampling (<60 accessions) or locus length (<400 bp) and used the remaining 1130 loci with an average length of 550 bp and had data for an average of 88 accessions. The coalescent simulations (Supplementary Figure S5) showed that the chance for a 600 bp locus to obtain three linked SNPs with a 10/96 frequency in a 96-accession population was less than 5%. So for the sequenced loci in the Nordborg Dataset, those having three (or more) linked SNPs, the frequency of which was equal to (or higher than) 10/96 was defined as dimorphic loci. In total, 307 (27.2% = 307/1130) dimorphic loci were identified, and four of those (Magnus Nordborg Derived Dimorphism loci, MND1–4) were randomly sampled to examine for evidence of indels.

**Figure 3:**
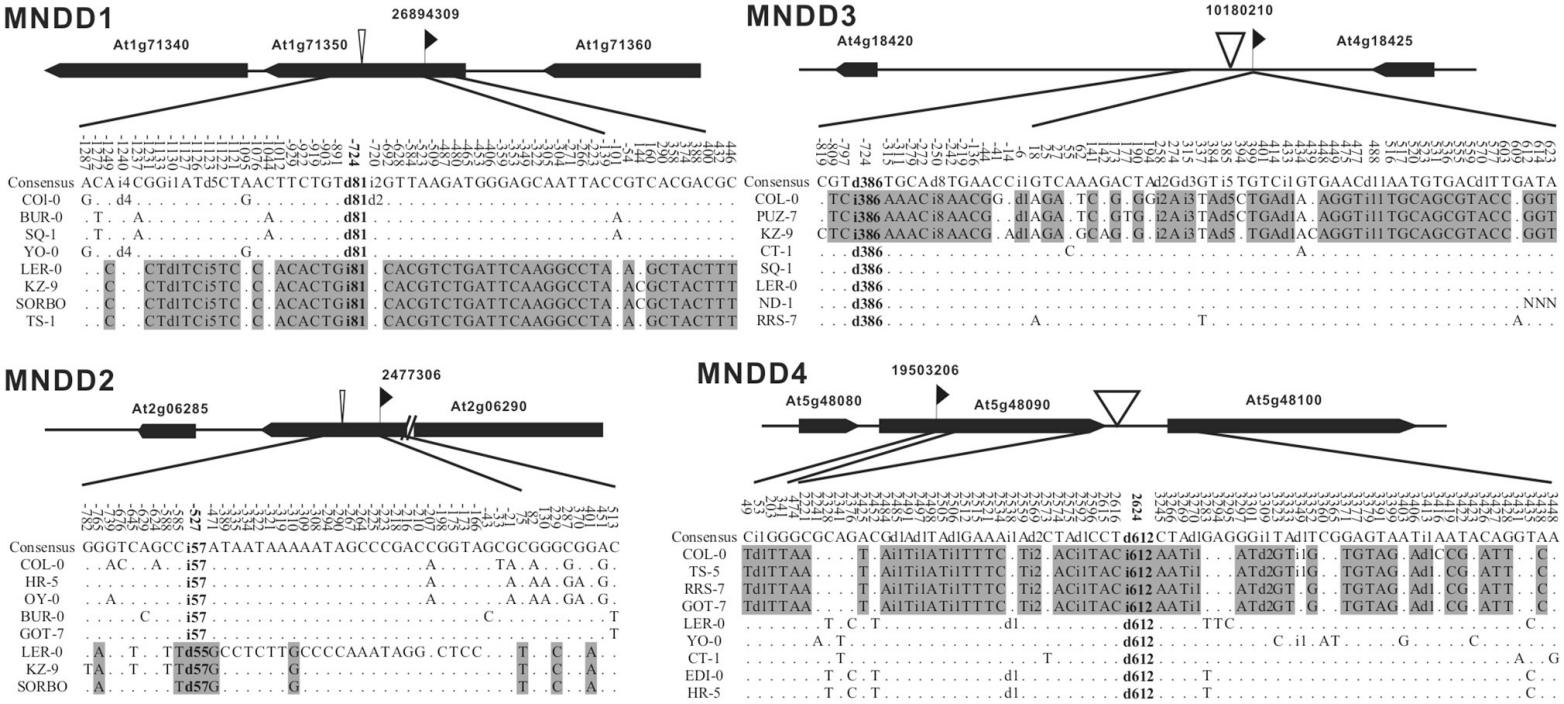
**Polymorphism around the selected dimorphic locus MND1–4**. The number represents the position relative to the first base pair of the sequenced regions in the Nordborg dataset (indicated by the flag). The extensions of the original dimorphic SNPs are marked by gray. The positions of identified indels are indicated by open triangles.

For each of our sampled loci, four accessions were randomly chosen from either of the two distinct haplotypes for further sequencing. The sequencing results revealed large indels in each of the four loci, located at 527, 724, 724, 2624 bp away from the original loci, and their sizes range from 57 to 612 bp (Figure 3). The indel -presence/-absence patterns in each of these loci are the same as the original dimorphisms. Although the location of the identified major indel in MND4 locus (2624 bp from the original dimorphic locus) is beyond the suggested extension in NRD loci (Figure 2B), the d_xy_ distribution still peaks around 100 bp from the indel. Noted that the original dimorphism is located in coding regions of the gene *At4g18420*, this long extension of dimorphism might reflect the selective forces exerting on the genes. Nevertheless, two of these four identified indels are smaller than the minimal size of major indels in RGD and NRD loci (100 bp). The finding of linked indels around sampled dimorphisms, together with the indel-centered dimorphisms shown above, clearly indicates a close association between indels and nucleotide dimorphisms, and that indels are playing a leading role in shaping this special polymorphic pattern in the genome.

### Nucleotide dimorphisms are affected by both genetic drift and selection

Nucleotide dimorphism could arise and be maintained by random genetic drift. The seemingly neutrally evolved dimorphisms can be seen at locus NRD1, 3, 7, 12, and 15, where almost all dimorphic sites and none-major indels reside in non-coding regions. For example, at locus RGD3, the blue-colored pattern in Supplementary Figure S5, a short and intact dimorphism (the total extension <0.79 kb), has 24 dimorphic sites. 23 of them are located in non-coding regions and one synonymous mutation is found in the coding region. Although ncRNAs and other regulatory elements could be present in non-coding regions, these dimorphisms located in non-coding regions are less likely maintained by selection, because we didn't find any overlap between 18 indel loci and the set of 2012 most highly conserved noncoding sequences (Haudry et al., 2013).

Meanwhile, natural selection may affect the maintenance of a dimorphism. The decay of d_xy_ is especially obvious in coding regions, e.g., 3′-1.4 kb region of locus RGD2 and 3′-3.0 kb region of locus RGD3 (Table 1). Furthermore, in the 307 dimorphic loci of the Nordborg data (Nordborg et al., 2005), the fixed substitutions in coding regions are significantly smaller than that in non-coding regions (3.5 vs. 6.3 sites per kb; *P* < 0.001, paired *t*-test, Supplementary Table S4), indicating an effect of purifying selection. On the other hand, the d_xy_ of junction regions in the five *R* genes (including locus *Rpm1* and *Rps5* Stahl et al., 1999; Tian et al., 2002) is obviously higher than those in the other 12 non-*R* loci with a single major indel (*P* < 0.001, paired *t*-test; Table 1 and Figures 2, 3A), indicating that balancing selection is working on these *R*-loci with present/absent polymorphism (Shen et al., 2006). The dimorphism is likely to be both stronger and wider in *R* genes because of the action of balancing selection. Thus, in addition to the influence from random drift, the extension of dimorphisms is affected by selective forces, negatively by purifying selection while positively by balancing selection.

### Controls: polymorphic pattern in random and neutral sequences

**Figure 4:**
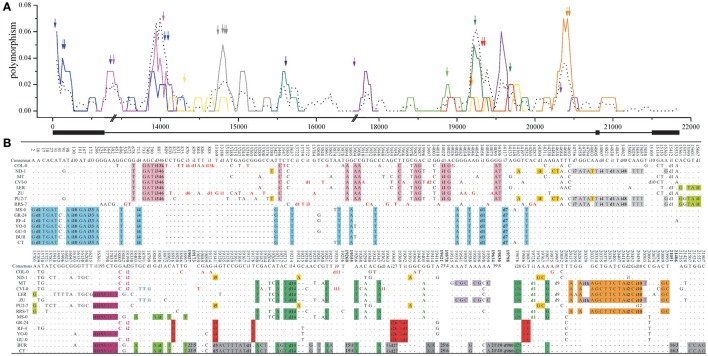
**Sliding window analysis of the nucleotide diversity (A)** and polymorphism **(B)** in long intergenic locus LI1. **(A)** The number (bp) on the horizontal axis represents the relative position in the long-intergenic sequences at each locus. The different-colored lines represent the sliding windows of the dimorphic diversity (d_xy_), and the dotted-black line stands for nucleotide diversity (π). The double slash (//) indicates a large insertion in one accession. Indels are marked as arrows, and lack boxes represent coding regions. **(B)** The i33 or d14 stands for a 33-bp insertion or 14-bp deletion. The different colored mutations are various indel-linked substitutions. And the bolded numbers represent the omitted polymorphic sites as 20/4 (20 substitutions and four indels were omitted in the region between the bolded positions), for example.

To confirm that the association between indels and dimorphisms revealed by our sampled indels or dimorphic loci are general examples of the polymorphic pattern in *Arabidopsis*, three sets of data were used as controls. First, four long intergenic regions (IL1–4; 8.2–11.4 kb long) were sampled to represent loci under neutral evolution. These long intergenic regions contain a full spectrum of non-selected indels and also should minimize the possible influence of selective forces, thus serving as controls. The four loci show the common existence of dimorphisms and a close association between the peaks of substitutions and indels (colored arrows) by sliding window analysis (Figure 4A and Supplementary Figure S5). If the nucleotide substitutions, which uniquely correspond to a present/absent pattern of indel(s) and are within 1 kb distance to any of these linked-indels, are defined as indel-linked mutations (the colored mutations), the nucleotide polymorphisms can be visually dissected into individual lineages (different dimorphisms). Then, 86.3% of indels (139 out of the total 161 indels) have the linked-substitutions, and 39 different dimorphic patterns are identified among these 139 indels (the different colored patterns in Figure 4B and Supplementary Figure S7). Similarly, 69.6% substitutions (498 out of the total 716 SNPs) have linked indels in their 1-kb flanking regions.

**Figure 5:**
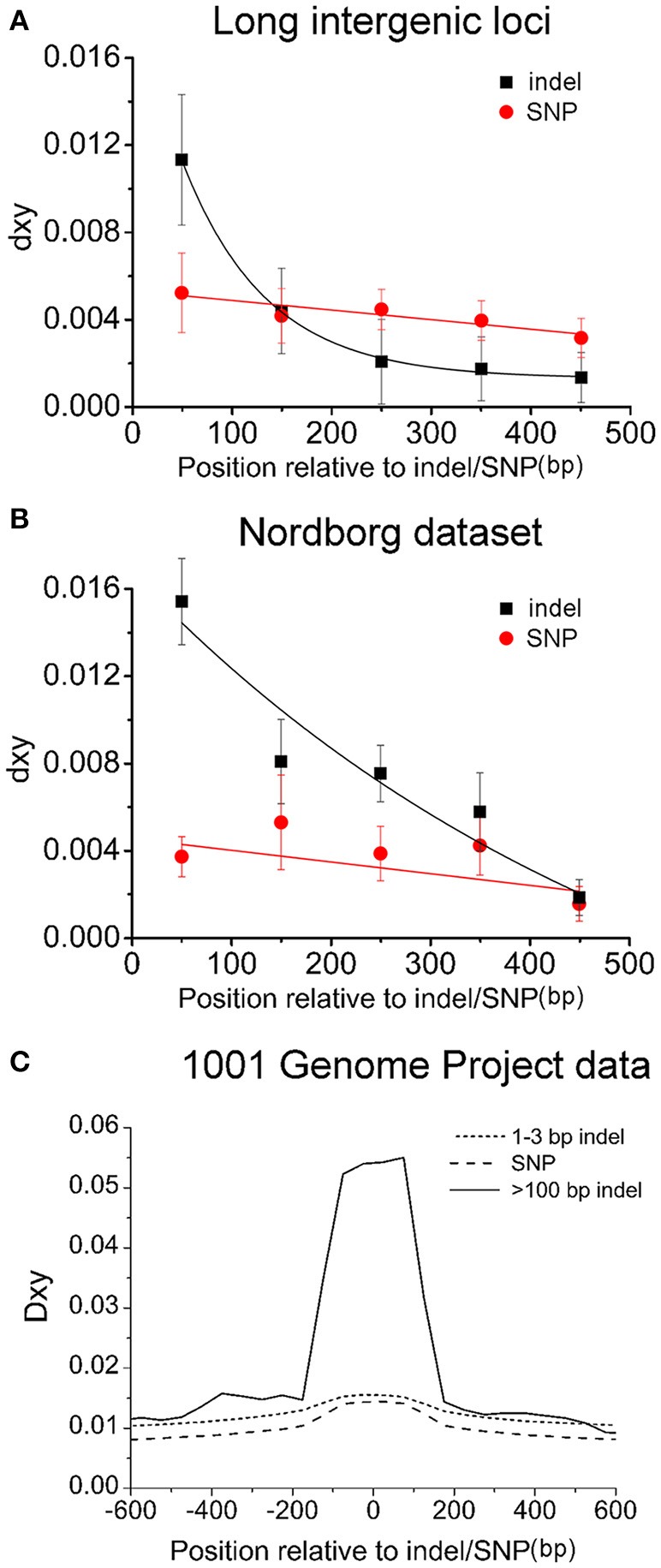
**Indel-centered distribution of nucleotide substitutions around indels in long intergenic regions (A)**, the Nordborg dataset **(B)** and the 1001 genome project **(C)**. In A, the balck-filled squares represent 103 indels within 39 different-colored (dimorphic) patterns and the red-filled circles stand for the sampled 39 SNPs, each of which is located at the closest position to the center point of a dimorphic pattern. **(B)** Relationship between distance from the first encountered indel or SNP at a locus and the corresponding d_xy_ in the Nordborg data. The standard error of mean (SEM) was denoted as error bar for each point. **(C)** Nucleotide polymorphism between insertion-present and –absent accessions around large indels (>100 bp), small indels (1–3 bp), and SNPs sampled from the genomic sequences of the 81 accessions produced by the 1001 Genome Project.

Remarkably in the long intergenic loci far from genes, compound dimorphisms and the high levels of nucleotide diversity or d_xy_ are common (Figure 4A and Supplementary Figure S7). There are many dimorphic patterns (i.e., the mutations without colored-background) and many high peaks of dimorphic diversity (d_xy_). For example, there are about 20 peaks of which d_xy_are >0.04, 13 peaks >0.06 and two peaks >0.20. Roughly every 1 or 2 kb region contains a highly divergent region. All these peaks, except two, are closely associated (within 100 bp) with corresponding indels. This association, demonstrated by d_xy_ sliding window analysis of the non-selected indels in non-coding regions, suggests that indel-linked substitutions could be accumulated in regions under relaxed selection and that dimorphisms might arise in the absence of any selective force. Figure 5A further shows a negative correlation between the indel-linked mutations (d_xy_) and the distance to the corresponding indel (*R*^2^ = 0.9995, *P* < 0.0001, one-phase exponential decay). To rule out the possibility that this indel-centered distribution is from the auto-correlation of the polymorphic sites, for each of the 39 identified dimorphisms, the SNP site closest to the central point of the extension of the dimorphism was set as the control polymorphic site for the indels. d_xy_ around those control SNPs was calculated accordingly. This analysis reveals that the d_xy_ around indels is higher compared to the d_xy_ around those control SNPs, and drops more rapidly (Figure 5A). This indicates that indels may play an essential role in the occurrence of a lineage and that the close association to dimorphism is specific to indels.

The second set of control is from the Nordborg Dataset (Nordborg et al., 2005) to examine dimorphic patterns of nucleotide polymorphism at randomly selected loci. In this data, there were 119 loci, which met the criteria used for picking non-*R* loci (diversity 0.01–0.05 between Col-0 and L*er*-1), with 66 loci having indel polymorphisms while the other 53 not. For the 66 indel containing loci (referred to MND indel loci here), the first encountered indel (differentiated between Col-0 and L*er*-1) in the alignment was picked and then the sequenced accessions were classified into two groups according to the presence or absence of the picked indel. The d_xy_ contributed by fixed SNPs between those two distinct haplotypes (also linked to the picked indel) was calculated. For the remaining 53 no-indel loci (referred to as MND SNP loci), the first encountered SNP in the alignment was picked, and the d_xy_ around was calculated accordingly to serve as a control (Figure 5B). The d_xy_ decays with increasing distance around both indels and control SNPs. However, the d_xy_ in the first 100 bp window for indels is significantly larger than that for control SNPs (1.53 and 0.36, *P* < 0.0001, paired *t*-test), and the average linked SNPs in the 500 bp region are 3.7 per indel locus, twice the number (1.7) per MND SNP locus. In addition, the associated dimorphic pattern (with fixed substitutions ≥3) was found in 24.5% of MND SNP loci (13/53), much less frequently than 54.5% (36/66) in MND indel loci (*P* < 0.001, chi-square test). Such differences remained when the analysis was restricted to the subset of loci containing only noncoding regions (Supplementary Figure S8). Thus, compared to MND SNP loci, the association between indel and dimorphism is much stronger in MND indel loci.

Thirdly, the complete genomic sequences of 81 *Arabidopsis* accessions produced by 1001 genome project (Cao et al., 2011) (http://www.1001genomes.org/) were examined to confirm this correlation between indels and their linked dimorphic SNPs. In total, 82 large indels with high quality sequence data (>100 bp etc., similar criteria as NRD loci, Methods for detail) were picked and further analyzed. SNPs and small indels (1–3 bp) picked according to the same criteria were served as controls. In the 82 large indel loci, the fixed nucleotide diversity between the indel-present and -absent accessions peaked within 100 bp from the deletion junction, and decayed sharply, which was not observed around SNPs or 1–3 indels (Figure 5C). This genome wide analysis confirmed what we observed from a smaller pool of indels: indel-centered non-random nucleotide dimorphisms are present around indels.

## Discussion

Our study revealed that a dimorphic pattern with highly divergent spots is present around 18 sampled indels and, conversely that indels are associated with four known dimorphic loci. For all these loci, the dimorphic haplotypes always correspond to the indel-present/-absent patterns, and the peaks of nucleotide diversities between the two divergent haplotypes are closely associated with these indels. The indel-centered distribution of linked nucleotide dimorphism is further confirmed by the long intergenic sequences and other two independently generated large genome datasets. There exists a close association between indels and dimorphisms or highly divergent spots. Thus, all the observations fit the expectations of indel-associated polymorphism model.

The close association of indels to their corresponding dimorphisms and the indel-centered distribution of d_xy_ suggest a mechanism linking indels and highly divergent spots. Indels could locally reduce recombination and indels are known to produce topological constraints for homologous pairing (Novitski and Braver, 1954; Grell, 1962; Hammarlund et al., 2005) which result in the reduced frequency of recombination. The suppression of recombination allows genetic isolation of the two haplotypes. Given enough time, mutations accumulate in each haplotype and leads to an indel-linked dimorphism with high divergence. In addition, the increased mutation rate surrounding indels (Tian et al., 2008; Conrad et al., 2010a,b; De and Babu, 2010; McDonald et al., 2011) could also accelerate the accumulation of dimorphic substitutions. Thus, nucleotide polymorphisms, resulting from point mutations, could be maintained in the deletion junction regions between haplotypes. When far away from either side of the unpaired insertion loops during meiosis, the region is less affected and is expected to exchange sequence more freely.

Our results are consistent with a model in which an indel could initiate a local isolation in the surrounding DNA. However, the association between indel and local isolation does not preclude other possibilities, such that SNPs induce local isolation as well. One alternative we considered is the possibility of strengthened isolation caused by both indel and SNP. A suppressed recombination has been repeatedly reported in the divergent sequences (Datta et al., 1997; Opperman et al., 2004), indicating that many SNPs alone can cause local isolation. The results from the sampling of SNPs in the Nordborg data demonstrated that a higher level of divergence is present surrounding these SNPs, although the level is only about one-fourth of that caused by indels (Figure 5B). Thus, we have good reason to assume that an indel could initiate an independent local isolation but a single SNP could not until many SNPs have been accumulated. When a region has both indels and SNPs, they could mutually strengthen the isolation effect. Meanwhile, our model doesn't preclude that distinct haplotypes could arise by the frequency-dependant selection or by the fusion of allopatric populations, which could contribute to the dimorphic patterns. However, our investigation on the fixed nucleotide diversity around indels showed that the locally- and commonly-occurring genetic isolation plays a key role in creating dimorphism and in shaping genome evolution.

Our indel-associated polymorphism model also predicts that the isolation effect caused by different indels should be independent if these indels are located distantly. This prediction was examined both directly and indirectly. First, if there is an independent effect, the different or distantly-located indels will generate different patterns of dimorphism around these indels. The different patterns around the 18 sampled indels and the well-matching patterns of mutation sites to multiple indels at a locus suggest that the isolation effects produced by indels are independent and that each indel induced its own nucleotide polymorphic pattern. In addition, the short extension of a dimorphism and the rapid decay of d_xy_ in dimorphic loci (Figure 5) suggest that the nucleotide dimorphisms occur independently. Furthermore, the increase of d_xy_ around the minor indels, located a short distance to the major indel, indicates that the influence of the minor indel on its surrounding regions is independent, at least partly, from that of major indels. These observations suggest that the indel-associated isolation exists locally and independently from indel to indel, consistent to the observation that there is no genome-wide dimorphic pattern (Du et al., 2008).

Given that indels are indeed associated with local genetic isolation, and mutation, an indel-centered distribution of d_xy_ is expected, particularly in the neutrally-evolved regions. Indeed, an extremely high value of correlation coefficient (*R*^2^ = 0.9995, *P* < 0.01) between d_xy_ and distance to indel, and a high proportion of indel-linked substitutions (69.6%) are present in the long intergenic regions (Figure 5A). On the other hand, a rapid decay of d_xy_ in coding regions is expected, because the indel-associated substitutions are detrimental in general. In fact, this phenomenon is repeatedly observed in coding regions in this study, which suggests that the deleterious mutations associated with indels are quickly removed in coding regions. These results indicate that the variation at the level of nucleotide diversity could be determined by the random occurrence and removal of indels.

Our indel-associated local isolation-mutation model predicts a higher d_xy_ around an indel when the indel is older. The d_xy_ is indeed higher (0.0710) in the first 100 bp around the indels of *R*-genes (Figure 2B) than around the other indels (0.0225). These *R*-genes, supposedly under balancing selection, are millions of years old (Stahl et al., 1999; Tian et al., 2002; Shen et al., 2006). Compared with *R*-genes, the other sampled indels were selected from those with nucleotide diversity 0.01–0.05 in the flanking regions between Col-0 and L*er*. Those indels were supposed to be younger than *R*-genes but older than the indels in the long intergenic regions, in which the average d_xy_ in the first 100 bp region is only 0.0113. These results are consistent with a neutral process of dimorphic-site fixation, observed from genome-wide analysis (Du et al., 2008).

The long intergenic region can serve as a control since it is thought to be evolving neutrally and has a full spectrum of indels that are not sampled by our established criteria. The four long-intergenic regions exhibit the common existence of the multiple dimorphisms. The sequence alignments and sliding window analyses (Figure 4A and Supplementary Figure S7) demonstrate the close association between d_xy_ and indels (Figure 5A) and the short extension (e.g., the different colored lines in Figure 5A), which show the independent and local effect of indel-associated isolation. Furthermore, the indel-linked mutations account for 69.6% of substitutions. The control sequences clearly show that isolation-associated nucleotide variation is common and that indel-associated genetic isolation might be a common mechanism in neutrally evolved regions.

Our studies reveal a close association between indels and nucleotide dimorphism in *A. thaliana*. We propose an indel-associated polymorphism model stating that indels are important for the maintenance of the nucleotide dimorphism/polymorphism in the population. Each indel is an “isolator or maintainer” of genetic variation, creates a propagation of “diversification front” (Vetsigian and Goldenfeld, 2005), which allows point difference to build up in the region flanking the indel, and eventually could cause the globe divergence of genome sequences. This is a more parsimonious explanation for the origin and maintenance of dimorphisms than those based on some form of frequency-dependent selection, which has often been invoked to explain dimorphism evolution. Our study suggests that the role played by indels in maintenance of genetic variation might be far more important than previously believed.

## Author contributions

CG, JD, RM, TG, and DT: Wrote the main text; CG, LW, and TG: Prepared the figures and tables; DT, SY, and TG: Designed the project; CG, JD, LW, and TG: Did the experiments and analysis; CG and JD: Contributed equally to this work. All authors reviewed the manuscript.

### Conflict of interest statement

The authors declare that the research was conducted in the absence of any commercial or financial relationships that could be construed as a potential conflict of interest.
