# Supplementary material for: Insertions/Deletions-Associated Nucleotide Polymorphism in Arabidopsis thaliana
Source: Front Plant Sci. 2016 Nov 30;7:1792. doi: 10.3389/fpls.2016.01792 (PMC5127803; doi:10.3389/fpls.2016.01792)
Supplement: Supplementary file 1 [file Table1.DOCX]

**Supplementary Table S1.** Features of the 764 indels between the accession Columbia (Col-0) and Landsberg *erecta* (L*er*) identified by Jander et al. (2002; [http://www.arabidopsis.org/ Cereon](http://www.arabidopsis.org/%20Cereon)). These indels are assumed to be insertions in Col-0 relative to L*er*. “N” means no indel, the zero distance between the given prefix and suffix sequences. “M” means multiple hits of the given prefix and suffix sequences, the unclear indel. “R” means redundancy, where the suggested indel is actually the same as another annotated indel and only one of them is selected. “T” means true indel.

|  | chr | BAC Name | Left | Right | indel Size |  | chr | BAC Name | Left | Right | Indel Size |
| --- | --- | --- | --- | --- | --- | --- | --- | --- | --- | --- | --- |
| N | 1 | T20M3 | 2322 | 2323 | 0 | N | 3 | MAL21 | 24378 | 24379 | 0 |
| N | 1 | T28P6 | 28619 | 28620 | 0 | N | 3 | T6H20 | 62583 | 62584 | 0 |
| N | 1 | F3N23 | 77354 | 77355 | 0 | N | 4 | T5L23 | 48137 | 48138 | 0 |
| N | 2 | T4E14 | 107248 | 107249 | 0 | N | 4 | FCA8 | 176912 | 176913 | 0 |
| N | 2 | T12J2 | 3296 | 3297 | 0 | N | 4 | FCA9 | 38913 | 38914 | 0 |
| N | 2 | T16B14 | 7491 | 7492 | 0 | N | 5 | K3K3 | 53868 | 53869 | 0 |
| N | 2 | F13K3 | 72110 | 72111 | 0 | N | 5 | MSL3 | 55997 | 55998 | 0 |
| N | 3 | F28J7 | 72695 | 72696 | 0 | N | 5 | K8K14 | 51306 | 51307 | 0 |
| N | 3 | F17A17 | 114550 | 114551 | 0 |  |  |  |  |  |  |
| M | 1 | F1N21 | 104981 | 105105 | 123 | M | 1 | T24D18 | 62106 | 68922 | 6815 |
| M | 1 | F5A8 | 100355 | 100479 | 123 | M | 2 | F23H14 | 22932 | 28384 | 5451 |
| M | 1 | F5I14 | 29801 | 32059 | 2257 | M | 3 | F10A16 | 19240 | 22021 | 2780 |
| M | 1 | T28P6 | 32475 | 35345 | 2869 | M | 4 | F28J12 | 31521 | 34193 | 2671 |
| M | 1 | T22I11 | 25184 | 28287 | 3102 | M | 5 | F5H8 | 39890 | 42824 | 2933 |
| M | 1 | F7G19 | 24028 | 29698 | 5669 | M | 5 | MXF12 | 1931 | 4947 | 3015 |
| M | 1 | T6H22 | 23185 | 29990 | 6804 |  |  |  |  |  |  |
| R | 1 | T1N6 | 41645 | 41748 | 102 | R | 3 | F18L15 | 73195 | 73600 | 404 |
| R | 1 | T5M16 | 45657 | 45773 | 115 | R | 3 | F13I12 | 127760 | 128221 | 460 |
| R | 1 | F14J16 | 14254 | 14396 | 141 | R | 3 | F13I12 | 127760 | 128221 | 460 |
| R | 1 | F11O6 | 82737 | 82894 | 156 | R | 3 | MZN24 | 4194 | 4908 | 713 |
| R | 1 | T2E12 | 26258 | 26419 | 160 | R | 3 | T21J18 | 56299 | 57022 | 722 |
| R | 1 | F19K6 | 1423 | 1598 | 174 | R | 3 | T21J18 | 3700 | 4473 | 772 |
| R | 1 | F27J15 | 58886 | 59093 | 206 | R | 3 | T21J18 | 3700 | 4473 | 772 |
| R | 1 | T26F17 | 65258 | 65495 | 236 | R | 3 | T8P19 | 90816 | 91589 | 772 |
| R | 1 | T26F17 | 65260 | 65497 | 236 | R | 3 | F12A12 | 21719 | 22527 | 807 |
| R | 1 | T26F17 | 65260 | 65497 | 236 | R | 3 | MIF6 | 4626 | 5492 | 865 |
| R | 1 | T26J14 | 1350 | 1619 | 268 | R | 3 | F18B3 | 12987 | 13992 | 1004 |
| R | 1 | T3F24 | 64773 | 65066 | 292 | R | 3 | T3A5 | 76181 | 77186 | 1004 |
| R | 1 | F1N21 | 104990 | 105303 | 312 | R | 3 | T3A5 | 42739 | 43756 | 1016 |
| R | 1 | F16M19 | 33989 | 34332 | 342 | R | 3 | MIL15 | 62234 | 63377 | 1142 |
| R | 1 | F17J6 | 1109 | 1599 | 489 | R | 3 | T8P19 | 54336 | 55482 | 1145 |
| R | 1 | T26F17 | 71109 | 71656 | 546 | R | 3 | MFE16 | 31985 | 33179 | 1193 |
| R | 1 | T2E6 | 47564 | 48161 | 596 | R | 3 | MQD17 | 10797 | 12019 | 1221 |
| R | 1 | T16N11 | 73501 | 74185 | 683 | R | 3 | T10K17 | 17453 | 19003 | 1549 |
| R | 1 | F4N2 | 30318 | 31078 | 759 | R | 3 | T10K17 | 17468 | 19018 | 1549 |
| R | 1 | F28H19 | 2762 | 3579 | 816 | R | 3 | T13O15 | 31998 | 33594 | 1595 |
| R | 1 | F28H19 | 19826 | 20647 | 820 | R | 3 | T8P19 | 72778 | 74418 | 1639 |
| R | 1 | F2J6 | 101865 | 102686 | 820 | R | 3 | T8P19 | 73035 | 74678 | 1642 |
| R | 1 | F2J6 | 101865 | 102686 | 820 | R | 3 | F18K10 | 75275 | 77231 | 1955 |
| R | 1 | T22C5 | 102330 | 103153 | 822 | R | 3 | F13I12 | 16578 | 18706 | 2127 |
| R | 1 | T22C5 | 99063 | 99969 | 905 | R | 3 | F10A16 | 19245 | 22026 | 2780 |
| R | 1 | T21F11 | 65458 | 66452 | 993 | R | 3 | F4P12 | 17351 | 20334 | 2982 |
| R | 1 | F4N2 | 27804 | 28849 | 1044 | R | 3 | T21J18 | 38450 | 41674 | 3223 |
| R | 1 | T22C5 | 93126 | 94398 | 1271 | R | 3 | F1P2 | 89198 | 93127 | 3928 |
| R | 1 | F28L5 | 10198 | 11470 | 1271 | R | 3 | F26O13 | 30678 | 35715 | 5036 |
| R | 1 | F10B6 | 12187 | 13473 | 1285 | R | 3 | MOA2 | 43432 | 49707 | 6274 |
| R | 1 | F11O6 | 90328 | 92025 | 1696 | R | 3 | MIG10 | 8862 | 15485 | 6622 |
| R | 1 | T1E4 | 2349 | 4101 | 1751 | R | 3 | MOD1 | 949 | 9097 | 8147 |
| R | 1 | T2E6 | 17327 | 19905 | 2577 | R | 3 | F11C1 | 2484 | 12962 | 10477 |
| R | 1 | F3M18 | 21293 | 23958 | 2664 | R | 3 | F3L24 | 5004 | 15485 | 10480 |
| R | 1 | F21H2 | 34125 | 36939 | 2813 | R | 3 | MFE16 | 16468 | 28746 | 12277 |
| R | 1 | T22I11 | 25247 | 28349 | 3101 | R | 3 | MZN24 | 42367 | 57977 | 15609 |
| R | 1 | T22I11 | 25184 | 28287 | 3102 | R | 4 | F10M10 | 10542 | 10644 | 101 |
| R | 1 | F24B9 | 32257 | 35375 | 3117 | R | 4 | F10M10 | 9888 | 9995 | 106 |
| R | 1 | F19K16 | 36776 | 41548 | 4771 | R | 4 | C17L7 | 29027 | 29139 | 111 |
| R | 1 | F2J6 | 72103 | 81241 | 9137 | R | 4 | C6L9 | 84774 | 84886 | 111 |
| R | 1 | T3F24 | 36749 | 47227 | 10477 | R | 4 | C6L9 | 84775 | 84887 | 111 |
| R | 1 | T2E6 | 43730 | 68815 | 25084 | R | 4 | F28A21 | 840 | 958 | 117 |
| R | 2 | T3B23 | 18158 | 18405 | 246 | R | 4 | T26N6 | 97214 | 97333 | 118 |
| R | 2 | F25P17 | 92834 | 93095 | 260 | R | 4 | T13D4 | 2313 | 2509 | 195 |
| R | 2 | F11F19 | 46702 | 46974 | 271 | R | 4 | F9H3 | 94813 | 95029 | 215 |
| R | 2 | MHK10 | 73375 | 73679 | 303 | R | 4 | T24G23 | 27913 | 28138 | 224 |
| R | 2 | T3P4 | 10253 | 10649 | 395 | R | 4 | F10M10 | 10173 | 10432 | 258 |
| R | 2 | F6P23 | 2909 | 3336 | 426 | R | 4 | C17L7 | 170925 | 171211 | 285 |
| R | 2 | T22C12 | 40186 | 40646 | 459 | R | 4 | F13M23 | 22399 | 22689 | 289 |
| R | 2 | F28N16 | 13866 | 14327 | 460 | R | 4 | F4H6 | 14238 | 14531 | 292 |
| R | 2 | F27L4 | 69471 | 70335 | 863 | R | 4 | T3E15 | 108395 | 108692 | 296 |
| R | 2 | F7O24 | 16918 | 17902 | 983 | R | 4 | F13M23 | 84908 | 85318 | 409 |
| R | 2 | T1E2 | 92763 | 93840 | 1076 | R | 4 | T2H3 | 22304 | 23289 | 984 |
| R | 2 | T2N18 | 70902 | 72145 | 1242 | R | 4 | F7L13 | 895 | 2226 | 1330 |
| R | 2 | T10J7 | 45424 | 46702 | 1277 | R | 4 | T9A4 | 33704 | 35616 | 1911 |
| R | 2 | T26I20 | 3236 | 4546 | 1309 | R | 4 | T2H3 | 14619 | 16706 | 2086 |
| R | 2 | F7O24 | 1889 | 4081 | 2191 | R | 4 | T2H3 | 14622 | 16709 | 2086 |
| R | 2 | F23H14 | 61687 | 64076 | 2388 | R | 4 | C17L7 | 12127 | 14279 | 2151 |
| R | 2 | F23H14 | 13133 | 16333 | 3199 | R | 4 | C6L9 | 67875 | 70027 | 2151 |
| R | 2 | F23M2 | 99196 | 102559 | 3362 | R | 4 | C6L9 | 67869 | 70021 | 2151 |
| R | 2 | F23H14 | 22932 | 28384 | 5451 | R | 4 | T6K22 | 68487 | 71739 | 3251 |
| R | 2 | T3P4 | 9772 | 15484 | 5711 | R | 4 | F20D10 | 135420 | 139403 | 3982 |
| R | 2 | F23H14 | 28142 | 42196 | 14053 | R | 4 | F19F18 | 82499 | 87322 | 4822 |
| R | 3 | F24B22 | 78100 | 78202 | 101 | R | 4 | F18E5 | 86261 | 91093 | 4831 |
| R | 3 | MOD1 | 36068 | 36175 | 106 | R | 4 | T8A17 | 50712 | 55593 | 4880 |
| R | 3 | MOD1 | 66731 | 66853 | 121 | R | 5 | F3F24 | 103924 | 104086 | 161 |
| R | 3 | MOD1 | 51860 | 52023 | 162 | R | 5 | MNJ8 | 8489 | 9013 | 523 |
| R | 3 | F15G16 | 77182 | 77369 | 186 | R | 5 | F7N22 | 104515 | 105360 | 844 |
| R | 3 | T12H1 | 4191 | 4434 | 242 | R | 5 | F7N22 | 104515 | 105360 | 844 |
| R | 3 | T11I18 | 1839 | 2121 | 281 | R | 5 | F7N22 | 107727 | 108632 | 904 |
| R | 3 | T21J18 | 33634 | 33928 | 293 | R | 5 | F7N22 | 107727 | 108632 | 904 |
| R | 3 | F12A12 | 11163 | 11469 | 305 | R | 5 | T21B4 | 16995 | 21228 | 4232 |
| R | 3 | F18L15 | 98247 | 98553 | 305 | R | 5 | F15F15 | 22177 | 26936 | 4758 |
| R | 3 | F12A12 | 11163 | 11469 | 305 | R | 5 | K21C13 | 15972 | 28250 | 12277 |
| R | 3 | T26I12 | 34940 | 35329 | 388 |  |  |  |  |  |  |
| T | 3 | F24B22 | 78100 | 78202 | 101 | T | 4 | C17L7 | 29026 | 29138 | 111 |
| T | 4 | F28A23 | 17118 | 17220 | 101 | T | 5 | K22F20 | 24796 | 24909 | 112 |
| T | 1 | T1N6 | 41645 | 41748 | 102 | T | 5 | MUL8 | 49064 | 49179 | 114 |
| T | 3 | T9J14 | 79186 | 79291 | 104 | T | 1 | T5M16 | 45657 | 45773 | 115 |
| T | 5 | F18G18 | 45915 | 46020 | 104 | T | 4 | T13J8 | 70782 | 70898 | 115 |
| T | 2 | F7B19 | 20662 | 20769 | 106 | T | 5 | MTE17 | 15516 | 15633 | 116 |
| T | 3 | MOD1 | 36068 | 36175 | 106 | T | 4 | F28J12 | 101298 | 101416 | 117 |
| T | 4 | F28A23 | 17767 | 17874 | 106 | T | 4 | F4H6 | 36132 | 36251 | 118 |
| T | 1 | F22L4 | 29406 | 29514 | 107 | T | 5 | F14F18 | 8375 | 8495 | 119 |
| T | 3 | F4P13 | 56591 | 56703 | 111 | T | 3 | MOD1 | 66731 | 66853 | 121 |
| T | 2 | F12C20 | 11957 | 12082 | 124 | T | 1 | T8L23 | 15011 | 15147 | 135 |
| T | 3 | F17J16 | 82929 | 83056 | 126 | T | 4 | T26N6 | 46332 | 46469 | 136 |
| T | 1 | T20M3 | 60300 | 60428 | 127 | T | 1 | F12F1 | 23481 | 23619 | 137 |
| T | 2 | F7D8 | 21258 | 21386 | 127 | T | 2 | F15O11 | 11726 | 11865 | 138 |
| T | 4 | F28D6 | 21366 | 21494 | 127 | T | 3 | MIE1 | 1113 | 1252 | 138 |
| T | 2 | MHK10 | 70550 | 70681 | 130 | T | 1 | T6H22 | 86305 | 86447 | 141 |
| T | 5 | MJJ3 | 59262 | 59393 | 130 | T | 4 | F4B14 | 15097 | 15241 | 143 |
| T | 2 | F4L23 | 60680 | 60812 | 131 | T | 3 | T5N23 | 50845 | 50993 | 147 |
| T | 2 | T10J7 | 61477 | 61609 | 131 | T | 2 | T10J7 | 77396 | 77545 | 148 |
| T | 5 | K1O13 | 21924 | 22056 | 131 | T | 2 | F4I1 | 52759 | 52909 | 149 |
| T | 2 | F3G5 | 102746 | 102898 | 151 | T | 3 | MOD1 | 51860 | 52023 | 162 |
| T | 1 | F15H11 | 92082 | 92236 | 153 | T | 1 | F13O11 | 89430 | 89595 | 164 |
| T | 4 | F6N15 | 59658 | 59813 | 154 | T | 4 | T15B16 | 71216 | 71382 | 165 |
| T | 1 | T20M3 | 60799 | 60955 | 155 | T | 2 | F13H10 | 64581 | 64748 | 166 |
| T | 4 | FCA6 | 49284 | 49440 | 155 | T | 1 | F21B7 | 48065 | 48233 | 167 |
| T | 1 | F21H2 | 10589 | 10746 | 156 | T | 2 | F2G1 | 8349 | 8517 | 167 |
| T | 3 | MJL12 | 62943 | 63100 | 156 | T | 1 | F21M12 | 25084 | 25253 | 168 |
| T | 5 | MJE4 | 64471 | 64631 | 159 | T | 2 | F28N16 | 21425 | 21594 | 168 |
| T | 1 | T22E19 | 3005 | 3166 | 160 | T | 1 | F15D2 | 14302 | 14475 | 172 |
| T | 5 | F3F24 | 103926 | 104088 | 161 | T | 1 | F19G10 | 30370 | 30543 | 172 |
| T | 2 | T4M8 | 18469 | 18642 | 172 | T | 2 | T1B8 | 25320 | 25498 | 177 |
| T | 2 | T16F16 | 65195 | 65369 | 173 | T | 4 | F2N1 | 59056 | 59237 | 180 |
| T | 3 | MXE2 | 34577 | 34751 | 173 | T | 5 | F14F18 | 8678 | 8860 | 181 |
| T | 4 | T12G13 | 89594 | 89768 | 173 | T | 2 | T20G20 | 8055 | 8239 | 183 |
| T | 1 | T20H2 | 59336 | 59511 | 174 | T | 3 | F15G16 | 77182 | 77369 | 186 |
| T | 1 | T1G11 | 74701 | 74876 | 174 | T | 2 | F26B6 | 53939 | 54128 | 188 |
| T | 1 | F6D8 | 103825 | 104000 | 174 | T | 5 | MIK22 | 38486 | 38675 | 188 |
| T | 1 | F6D8 | 114159 | 114334 | 174 | T | 4 | T17A2 | 68678 | 68874 | 195 |
| T | 1 | T20M3 | 60516 | 60692 | 175 | T | 5 | MAB16 | 66629 | 66827 | 197 |
| T | 5 | K10D11 | 896 | 1073 | 176 | T | 2 | F16G22 | 6529 | 6729 | 199 |
| T | 5 | F3F24 | 74704 | 74904 | 199 | T | 5 | K11J9 | 9281 | 9506 | 224 |
| T | 1 | F14M2 | 68309 | 68514 | 204 | T | 5 | MSN2 | 16640 | 16868 | 227 |
| T | 5 | MBB18 | 36078 | 36284 | 205 | T | 4 | T4I9 | 77491 | 77721 | 229 |
| T | 1 | F27J15 | 58886 | 59093 | 206 | T | 2 | T30B22 | 35073 | 35306 | 232 |
| T | 2 | T12H3 | 5816 | 6027 | 210 | T | 5 | MKD15 | 36857 | 37090 | 232 |
| T | 2 | F26C24 | 51410 | 51621 | 210 | T | 1 | T26F17 | 65258 | 65495 | 236 |
| T | 3 | T4P3 | 54161 | 54377 | 215 | T | 3 | K17E12 | 1630 | 1869 | 238 |
| T | 4 | F4C21 | 130088 | 130304 | 215 | T | 3 | T12H1 | 4191 | 4434 | 242 |
| T | 1 | T19E23 | 52829 | 53049 | 219 | T | 4 | T9A14 | 33769 | 34013 | 243 |
| T | 4 | F5K24 | 109185 | 109410 | 224 | T | 2 | F12C20 | 12181 | 12426 | 244 |
| T | 4 | T28D5 | 10239 | 10484 | 244 | T | 4 | F28A23 | 17330 | 17589 | 258 |
| T | 2 | T32F12 | 13888 | 14135 | 246 | T | 2 | F3L12 | 85552 | 85812 | 259 |
| T | 2 | T3B23 | 18158 | 18405 | 246 | T | 2 | F8D23 | 20962 | 21223 | 260 |
| T | 3 | F27H5 | 13453 | 13700 | 246 | T | 2 | T28I24 | 76586 | 76847 | 260 |
| T | 4 | F8D20 | 64807 | 65054 | 246 | T | 3 | MDJ14 | 28332 | 28594 | 261 |
| T | 3 | MSJ3 | 26620 | 26869 | 248 | T | 2 | F5H14 | 33505 | 33773 | 267 |
| T | 1 | F22O13 | 123996 | 124246 | 249 | T | 1 | T2E12 | 6845 | 7114 | 268 |
| T | 2 | T12H3 | 263 | 516 | 252 | T | 1 | T14L22 | 4017 | 4287 | 269 |
| T | 2 | F15A23 | 13179 | 13432 | 252 | T | 3 | F1I16 | 17857 | 18128 | 270 |
| T | 2 | T12H3 | 18520 | 18774 | 253 | T | 2 | F11F19 | 46705 | 46977 | 271 |
| T | 5 | MHK7 | 42278 | 42557 | 278 | T | 2 | T12H3 | 5650 | 5945 | 294 |
| T | 1 | F19K23 | 7010 | 7291 | 280 | T | 4 | T3E15 | 108395 | 108692 | 296 |
| T | 3 | F20H23 | 11551 | 11833 | 281 | T | 2 | T9I22 | 41039 | 41337 | 297 |
| T | 1 | F20P5 | 32573 | 32857 | 283 | T | 3 | T13J10 | 2851 | 3151 | 299 |
| T | 4 | T32N4 | 20373 | 20659 | 285 | T | 2 | F14N22 | 95403 | 95707 | 303 |
| T | 5 | K19E20 | 6109 | 6398 | 288 | T | 3 | F18B3 | 22227 | 22531 | 303 |
| T | 4 | F6I7 | 74722 | 75012 | 289 | T | 2 | T16I21 | 37360 | 37665 | 304 |
| T | 1 | T3F24 | 64773 | 65066 | 292 | T | 3 | F18L15 | 98247 | 98553 | 305 |
| T | 4 | T26N6 | 75321 | 75614 | 292 | T | 1 | F5A8 | 100157 | 100470 | 312 |
| T | 3 | T21J18 | 33634 | 33928 | 293 | T | 2 | T16B12 | 49438 | 49751 | 312 |
| T | 2 | T1O3 | 58915 | 59228 | 312 | T | 2 | T8O18 | 46583 | 46920 | 336 |
| T | 5 | K18P6 | 32444 | 32760 | 315 | T | 5 | T2L5 | 23865 | 24202 | 336 |
| T | 5 | MUB3 | 64554 | 64870 | 315 | T | 3 | T22K18 | 19689 | 20028 | 338 |
| T | 3 | F3L24 | 46046 | 46364 | 317 | T | 4 | F21P8 | 73835 | 74175 | 339 |
| T | 4 | F15P23 | 8746 | 9066 | 319 | T | 2 | T26I20 | 12482 | 12823 | 340 |
| T | 5 | MPH15 | 39170 | 39495 | 324 | T | 1 | F16M19 | 33989 | 34332 | 342 |
| T | 2 | F2I9 | 70714 | 71041 | 326 | T | 3 | K5K13 | 10255 | 10601 | 345 |
| T | 5 | MYH19 | 16711 | 17038 | 326 | T | 4 | T19J18 | 32761 | 33113 | 351 |
| T | 5 | MPL12 | 45568 | 45897 | 328 | T | 4 | T16H5 | 76567 | 76923 | 355 |
| T | 5 | T21B4 | 58743 | 59072 | 328 | T | 5 | F14I23 | 39764 | 40120 | 355 |
| T | 5 | T3P1 | 3663 | 4026 | 362 | T | 2 | F13D4 | 49299 | 49694 | 394 |
| T | 5 | K24M7 | 25955 | 26319 | 363 | T | 2 | T3P4 | 10253 | 10649 | 395 |
| T | 4 | F13C5 | 6153 | 6525 | 371 | T | 2 | F24C20 | 6056 | 6453 | 396 |
| T | 5 | MUL8 | 54594 | 54970 | 375 | T | 5 | MLN1 | 218 | 615 | 396 |
| T | 4 | F18F4 | 54451 | 54829 | 377 | T | 5 | MJC20 | 50949 | 51350 | 400 |
| T | 5 | MYJ24 | 42786 | 43166 | 379 | T | 4 | F20O9 | 9125 | 9527 | 401 |
| T | 4 | T4C9 | 33080 | 33461 | 380 | T | 5 | F3F24 | 38061 | 38464 | 402 |
| T | 5 | MPL12 | 74940 | 75326 | 385 | T | 3 | F18L15 | 73195 | 73600 | 404 |
| T | 3 | T26I12 | 34940 | 35329 | 388 | T | 4 | F24A6 | 24775 | 25185 | 409 |
| T | 5 | MJG14 | 42044 | 42437 | 392 | T | 4 | F11O4 | 46976 | 47389 | 412 |
| T | 2 | T13E11 | 8270 | 8691 | 420 | T | 3 | T21L8 | 9753 | 10214 | 460 |
| T | 2 | T2P4 | 9862 | 10283 | 420 | T | 5 | MBD2 | 65630 | 66092 | 461 |
| T | 2 | F12A24 | 89529 | 89956 | 426 | T | 4 | T10C21 | 24531 | 24998 | 466 |
| T | 5 | MJG14 | 63859 | 64308 | 448 | T | 3 | F28O9 | 25601 | 26078 | 476 |
| T | 1 | F1K23 | 71665 | 72115 | 449 | T | 1 | T17H3 | 12433 | 12912 | 478 |
| T | 4 | T25K17 | 60705 | 61162 | 456 | T | 2 | F25P17 | 42353 | 42841 | 487 |
| T | 2 | F15A23 | 19939 | 20399 | 459 | T | 1 | F11F12 | 76169 | 76659 | 489 |
| T | 5 | T2L5 | 29237 | 29697 | 459 | T | 3 | T21L8 | 60834 | 61336 | 501 |
| T | 1 | F10B6 | 68657 | 69118 | 460 | T | 2 | F22D22 | 27573 | 28082 | 508 |
| T | 2 | F28N16 | 13866 | 14327 | 460 | T | 1 | F12K21 | 13602 | 14116 | 513 |
| T | 3 | F3L24 | 9625 | 10139 | 513 | T | 2 | T11J7 | 55693 | 56243 | 549 |
| T | 1 | F16P17 | 2593 | 3110 | 516 | T | 4 | FCA5 | 78233 | 78783 | 549 |
| T | 2 | T15J14 | 99981 | 100502 | 520 | T | 5 | F21J6 | 85201 | 85757 | 555 |
| T | 5 | MNJ8 | 8492 | 9016 | 523 | T | 4 | F9H3 | 70524 | 71086 | 561 |
| T | 2 | F11C10 | 55245 | 55779 | 533 | T | 1 | F20D23 | 59924 | 60487 | 562 |
| T | 1 | F24O1 | 29046 | 29582 | 535 | T | 5 | MRH10 | 56516 | 57079 | 562 |
| T | 3 | MXE2 | 79848 | 80391 | 542 | T | 5 | F5H8 | 50942 | 51506 | 563 |
| T | 5 | T20O7 | 9057 | 9600 | 542 | T | 5 | T25C13 | 101748 | 102318 | 569 |
| T | 1 | T26F17 | 71109 | 71656 | 546 | T | 2 | F7E22 | 56770 | 57349 | 578 |
| T | 2 | F18O19 | 7776 | 8326 | 549 | T | 3 | F18C1 | 54449 | 55033 | 583 |
| T | 1 | T2E6 | 47564 | 48161 | 596 | T | 3 | T22K18 | 7611 | 8260 | 648 |
| T | 3 | F24P17 | 32272 | 32883 | 610 | T | 2 | F23N11 | 28935 | 29601 | 665 |
| T | 2 | F13D4 | 46747 | 47370 | 622 | T | 2 | F28N16 | 11097 | 11764 | 666 |
| T | 4 | F4C21 | 120282 | 120908 | 625 | T | 4 | FCA7 | 64571 | 65244 | 672 |
| T | 1 | T9L6 | 17404 | 18031 | 626 | T | 1 | T16N11 | 73501 | 74185 | 683 |
| T | 1 | F23H11 | 135082 | 135709 | 626 | T | 1 | F1N21 | 12838 | 13525 | 686 |
| T | 4 | FCA6 | 133217 | 133844 | 626 | T | 3 | MLJ15 | 38655 | 39344 | 688 |
| T | 1 | F6D8 | 42397 | 43027 | 629 | T | 4 | FCA3 | 161391 | 162086 | 694 |
| T | 3 | MSA6 | 50972 | 51609 | 636 | T | 5 | MAB16 | 982 | 1683 | 700 |
| T | 4 | T7M24 | 41844 | 42491 | 646 | T | 3 | MZN24 | 4194 | 4908 | 713 |
| T | 3 | T21J18 | 56299 | 57022 | 722 | T | 4 | F18F4 | 17726 | 18525 | 798 |
| T | 5 | MJE4 | 50778 | 51518 | 739 | T | 1 | F14I3 | 48516 | 49318 | 801 |
| T | 2 | T25N22 | 17305 | 18055 | 749 | T | 3 | F12A12 | 21719 | 22527 | 807 |
| T | 3 | MKA23 | 14028 | 14779 | 750 | T | 1 | F19P19 | 38084 | 38893 | 808 |
| T | 1 | F4N2 | 30318 | 31078 | 759 | T | 2 | T16B12 | 55565 | 56374 | 808 |
| T | 2 | F26H11 | 46427 | 47194 | 766 | T | 1 | F28H19 | 2762 | 3579 | 816 |
| T | 1 | T7A14 | 41140 | 41912 | 771 | T | 1 | F28H19 | 19826 | 20647 | 820 |
| T | 3 | T8P19 | 90816 | 91589 | 772 | T | 5 | F5H8 | 32336 | 33157 | 820 |
| T | 3 | F10A16 | 23503 | 24279 | 775 | T | 1 | F21H2 | 43791 | 44614 | 822 |
| T | 3 | MPE11 | 81389 | 82178 | 788 | T | 1 | T22C5 | 102330 | 103153 | 822 |
| T | 5 | T31P16 | 26300 | 27128 | 827 | T | 3 | F26K24 | 16872 | 17742 | 869 |
| T | 4 | T3H13 | 50349 | 51193 | 843 | T | 4 | T10P11 | 11509 | 12381 | 871 |
| T | 5 | MSK10 | 15348 | 16193 | 844 | T | 5 | MBK20 | 75224 | 76105 | 880 |
| T | 4 | F11I11 | 42925 | 43772 | 846 | T | 1 | F23N19 | 24786 | 25677 | 890 |
| T | 4 | F13M23 | 40135 | 40985 | 849 | T | 4 | F6H8 | 40961 | 41853 | 891 |
| T | 4 | F6I7 | 92461 | 93311 | 849 | T | 1 | F15O4 | 117197 | 118090 | 892 |
| T | 3 | K7M2 | 53483 | 54342 | 858 | T | 1 | F8K4 | 108594 | 109497 | 902 |
| T | 2 | F26B6 | 121464 | 122328 | 863 | T | 5 | MSK10 | 12076 | 12981 | 904 |
| T | 3 | MIF6 | 4626 | 5492 | 865 | T | 1 | T22C5 | 99063 | 99969 | 905 |
| T | 1 | T28P6 | 55064 | 55933 | 868 | T | 5 | MJE4 | 58875 | 59783 | 907 |
| T | 2 | F26C24 | 46585 | 47494 | 908 | T | 1 | T28P6 | 42841 | 43809 | 967 |
| T | 5 | MKD15 | 45001 | 45916 | 914 | T | 1 | F20D23 | 23532 | 24504 | 971 |
| T | 4 | T3F12 | 46942 | 47862 | 919 | T | 2 | F26H11 | 2933 | 3917 | 983 |
| T | 5 | K15E6 | 44148 | 45069 | 920 | T | 5 | K21L13 | 25797 | 26781 | 983 |
| T | 2 | F19I3 | 30761 | 31684 | 922 | T | 4 | T2H3 | 22304 | 23289 | 984 |
| T | 1 | T5I8 | 15248 | 16194 | 945 | T | 1 | F5I6 | 79698 | 80692 | 993 |
| T | 1 | F26A9 | 87712 | 88662 | 949 | T | 5 | F15L12 | 40456 | 41455 | 998 |
| T | 5 | F21J6 | 78193 | 79146 | 952 | T | 1 | F19P19 | 71978 | 72980 | 1001 |
| T | 2 | T20K24 | 7165 | 8121 | 955 | T | 1 | F11F12 | 83787 | 84791 | 1003 |
| T | 4 | F11C18 | 24339 | 25301 | 961 | T | 3 | T3A5 | 76181 | 77186 | 1004 |
| T | 3 | T3A5 | 42739 | 43756 | 1016 | T | 4 | F25G13 | 91023 | 92100 | 1076 |
| T | 3 | F9F8 | 36849 | 37870 | 1020 | T | 4 | F4C21 | 86856 | 87934 | 1077 |
| T | 1 | F11P17 | 38880 | 39910 | 1029 | T | 3 | MLJ15 | 18069 | 19148 | 1078 |
| T | 1 | F3F20 | 86926 | 87964 | 1037 | T | 3 | F9F8 | 85582 | 86661 | 1078 |
| T | 1 | F4N2 | 27804 | 28849 | 1044 | T | 2 | F26H11 | 30773 | 31900 | 1126 |
| T | 5 | MMG4 | 973 | 2019 | 1045 | T | 4 | F17A8 | 76764 | 77891 | 1126 |
| T | 4 | F9F13 | 18398 | 19445 | 1046 | T | 5 | MQK4 | 49867 | 51003 | 1135 |
| T | 2 | T5I7 | 68292 | 69358 | 1065 | T | 3 | MIL15 | 62234 | 63377 | 1142 |
| T | 5 | MLF18 | 15200 | 16270 | 1069 | T | 3 | T8P19 | 54336 | 55482 | 1145 |
| T | 2 | F15K20 | 11420 | 12497 | 1076 | T | 5 | F11P10 | 8628 | 9791 | 1162 |
| T | 4 | T10C21 | 22646 | 23825 | 1178 | T | 2 | T1J8 | 127993 | 129236 | 1242 |
| T | 5 | MJE4 | 14071 | 15254 | 1182 | T | 4 | T26N6 | 29441 | 30693 | 1251 |
| T | 5 | MXA21 | 62317 | 63508 | 1190 | T | 4 | T28D5 | 39890 | 41142 | 1251 |
| T | 3 | MFE16 | 31985 | 33179 | 1193 | T | 5 | MBK23 | 76209 | 77461 | 1251 |
| T | 5 | MIK22 | 59933 | 61128 | 1194 | T | 5 | MUB3 | 75587 | 76847 | 1259 |
| T | 5 | MXC20 | 71879 | 73074 | 1194 | T | 1 | T22C5 | 93126 | 94398 | 1271 |
| T | 1 | T10O24 | 78837 | 80042 | 1204 | T | 2 | F23M2 | 116538 | 117816 | 1277 |
| T | 2 | T1O3 | 39383 | 40600 | 1216 | T | 5 | MHM17 | 10514 | 11795 | 1280 |
| T | 4 | F26P21 | 53893 | 55114 | 1220 | T | 1 | T5E21 | 81678 | 82964 | 1285 |
| T | 3 | MQD17 | 10797 | 12019 | 1221 | T | 4 | T4C9 | 34471 | 35761 | 1289 |
| T | 5 | K22F20 | 43886 | 45180 | 1293 | T | 3 | MJL12 | 72810 | 74187 | 1376 |
| T | 2 | F26C24 | 4198 | 5508 | 1309 | T | 4 | F24J7 | 76848 | 78232 | 1383 |
| T | 4 | T10I14 | 24157 | 25478 | 1320 | T | 2 | T16F16 | 11958 | 13356 | 1397 |
| T | 4 | F24G24 | 74189 | 75520 | 1330 | T | 5 | F14A1 | 54154 | 55584 | 1429 |
| T | 5 | MWD22 | 37105 | 38436 | 1330 | T | 3 | T10K17 | 17453 | 19003 | 1549 |
| T | 1 | T6L1 | 31756 | 33093 | 1336 | T | 3 | T10K17 | 17468 | 19018 | 1549 |
| T | 5 | T25O11 | 37085 | 38422 | 1336 | T | 1 | F1K23 | 39782 | 41339 | 1556 |
| T | 1 | T4O12 | 86558 | 87897 | 1338 | T | 2 | T9I22 | 44041 | 45624 | 1582 |
| T | 2 | T3A4 | 49973 | 51320 | 1346 | T | 4 | F9F13 | 99078 | 100666 | 1587 |
| T | 4 | T22F8 | 63136 | 64484 | 1347 | T | 3 | T22N4 | 8059 | 9655 | 1595 |
| T | 5 | MSK20 | 383 | 1992 | 1608 | T | 4 | F8D20 | 7121 | 8928 | 1806 |
| T | 1 | T17H3 | 15067 | 16684 | 1616 | T | 1 | F12P19 | 58726 | 60538 | 1811 |
| T | 3 | T8P19 | 72778 | 74418 | 1639 | T | 5 | T21B4 | 62768 | 64616 | 1847 |
| T | 3 | T8P19 | 73035 | 74678 | 1642 | T | 1 | F1I21 | 97450 | 99309 | 1858 |
| T | 2 | F13J11 | 32492 | 34157 | 1664 | T | 1 | F5O8 | 80265 | 82137 | 1871 |
| T | 1 | F21H2 | 18180 | 19877 | 1696 | T | 2 | F15N24 | 19108 | 20987 | 1878 |
| T | 4 | T24H24 | 4473 | 6185 | 1711 | T | 1 | F1K23 | 48413 | 50295 | 1881 |
| T | 1 | F14M2 | 100165 | 101917 | 1751 | T | 3 | F5N5 | 12970 | 14852 | 1881 |
| T | 1 | T24P13 | 63708 | 65473 | 1764 | T | 4 | F24G24 | 11325 | 13237 | 1911 |
| T | 2 | F14O4 | 9743 | 11509 | 1765 | T | 1 | F12K11 | 22313 | 24254 | 1940 |
| T | 5 | K16F13 | 13679 | 15622 | 1942 | T | 4 | T2H3 | 14619 | 16706 | 2086 |
| T | 3 | F13M14 | 27286 | 29242 | 1955 | T | 5 | MAB16 | 7115 | 9225 | 2109 |
| T | 4 | F21I2 | 25979 | 27938 | 1958 | T | 4 | FCA1 | 166359 | 168475 | 2115 |
| T | 5 | F18G18 | 78345 | 80311 | 1965 | T | 2 | F4I1 | 50472 | 52589 | 2116 |
| T | 2 | F4L23 | 36879 | 38858 | 1978 | T | 3 | F13I12 | 16578 | 18706 | 2127 |
| T | 4 | T10I14 | 58405 | 60385 | 1979 | T | 4 | T15B16 | 67396 | 69525 | 2128 |
| T | 1 | T19E23 | 69112 | 71107 | 1994 | T | 4 | C17L7 | 12121 | 14273 | 2151 |
| T | 4 | F23K16 | 62316 | 64312 | 1995 | T | 2 | F3K23 | 2000 | 4192 | 2191 |
| T | 5 | MJE7 | 66220 | 68261 | 2040 | T | 5 | MLF18 | 6327 | 8551 | 2223 |
| T | 2 | T4C15 | 8709 | 10787 | 2077 | T | 2 | T3F17 | 17313 | 19549 | 2235 |
| T | 5 | MBK5 | 43285 | 45541 | 2255 | T | 1 | F3M18 | 21293 | 23958 | 2664 |
| T | 4 | F24G24 | 92888 | 95225 | 2336 | T | 4 | F21C20 | 20338 | 23018 | 2679 |
| T | 2 | T1O24 | 55237 | 57616 | 2378 | T | 5 | MJJ3 | 8998 | 11690 | 2691 |
| T | 2 | F23H14 | 61687 | 64076 | 2388 | T | 4 | T24M8 | 60419 | 63135 | 2715 |
| T | 2 | T26C19 | 29134 | 31618 | 2483 | T | 2 | T20G20 | 47889 | 50640 | 2750 |
| T | 1 | F11O6 | 4773 | 7285 | 2511 | T | 2 | T15J14 | 40445 | 43215 | 2769 |
| T | 5 | F13G24 | 15834 | 18367 | 2532 | T | 1 | F11O6 | 106273 | 109087 | 2813 |
| T | 5 | MXM12 | 31239 | 33772 | 2532 | T | 5 | F21J6 | 87147 | 90021 | 2873 |
| T | 3 | F28D10 | 21142 | 23702 | 2559 | T | 3 | K7M2 | 57941 | 60820 | 2878 |
| T | 1 | T2E6 | 17327 | 19905 | 2577 | T | 2 | T12J2 | 50960 | 53849 | 2888 |
| T | 3 | MQC3 | 10207 | 13108 | 2900 | T | 2 | T10F5 | 47297 | 50500 | 3202 |
| T | 2 | F26C24 | 51057 | 53995 | 2937 | T | 4 | T19F6 | 33255 | 36460 | 3204 |
| T | 3 | T6D9 | 10635 | 13604 | 2968 | T | 3 | T21J18 | 38450 | 41674 | 3223 |
| T | 3 | F4P12 | 17351 | 20334 | 2982 | T | 2 | T20G20 | 92056 | 95308 | 3251 |
| T | 3 | T29H11 | 70308 | 73291 | 2982 | T | 4 | F18E5 | 5042 | 8294 | 3251 |
| T | 4 | T1P17 | 88342 | 91365 | 3022 | T | 4 | F1N20 | 74430 | 77700 | 3269 |
| T | 1 | T22I11 | 25247 | 28349 | 3101 | T | 2 | T10J7 | 28082 | 31445 | 3362 |
| T | 1 | F24B9 | 32257 | 35375 | 3117 | T | 4 | F11O4 | 6254 | 9656 | 3401 |
| T | 1 | F1O17 | 18748 | 21897 | 3148 | T | 5 | MWJ3 | 32864 | 36324 | 3459 |
| T | 2 | F23H14 | 13133 | 16333 | 3199 | T | 5 | F3F24 | 93087 | 96632 | 3544 |
| T | 1 | F20N2 | 43804 | 47389 | 3584 | T | 4 | F22I13 | 15386 | 19369 | 3982 |
| T | 1 | T23J18 | 38695 | 42296 | 3600 | T | 5 | K24M7 | 10944 | 15025 | 4080 |
| T | 1 | F24D7 | 23317 | 26947 | 3629 | T | 1 | F13A11 | 7328 | 11521 | 4192 |
| T | 1 | F7A19 | 104882 | 108512 | 3629 | T | 5 | F21A20 | 2686 | 6919 | 4232 |
| T | 5 | F21A20 | 42584 | 46221 | 3636 | T | 3 | T2O9 | 13419 | 17714 | 4294 |
| T | 2 | T29E15 | 75634 | 79409 | 3774 | T | 1 | F28J9 | 47766 | 52080 | 4313 |
| T | 4 | F28J12 | 30833 | 34615 | 3781 | T | 3 | F5E6 | 10969 | 15385 | 4415 |
| T | 5 | MCD7 | 43075 | 46865 | 3789 | T | 2 | F3L12 | 46798 | 51239 | 4440 |
| T | 1 | F3O9 | 61960 | 65798 | 3837 | T | 1 | F1N21 | 88375 | 92830 | 4454 |
| T | 3 | F1P2 | 89198 | 93127 | 3928 | T | 4 | F24A6 | 78683 | 83176 | 4492 |
| T | 4 | F7K2 | 22027 | 26552 | 4524 | T | 5 | MFB13 | 6223 | 11049 | 4825 |
| T | 2 | F4L23 | 92093 | 96678 | 4584 | T | 4 | F17L22 | 13212 | 18044 | 4831 |
| T | 3 | T23J7 | 52458 | 57050 | 4591 | T | 2 | T13C7 | 9514 | 14351 | 4836 |
| T | 5 | K2N11 | 24992 | 29624 | 4631 | T | 5 | MDK4 | 4772 | 9646 | 4873 |
| T | 2 | F5G3 | 77845 | 82586 | 4740 | T | 5 | T11H3 | 28193 | 33070 | 4876 |
| T | 5 | K21C13 | 38659 | 43407 | 4747 | T | 4 | F23J3 | 74768 | 79649 | 4880 |
| T | 5 | F15F15 | 22177 | 26936 | 4758 | T | 5 | MOK9 | 74920 | 79824 | 4903 |
| T | 1 | F19K16 | 36730 | 41503 | 4772 | T | 1 | T28P6 | 32353 | 37315 | 4961 |
| T | 4 | T28I19 | 10880 | 15703 | 4822 | T | 3 | MXE2 | 70353 | 75327 | 4973 |
| T | 3 | MAG2 | 27048 | 31872 | 4823 | T | 5 | T25C13 | 52625 | 57612 | 4986 |
| T | 3 | F26O13 | 30678 | 35715 | 5036 | T | 5 | MIK22 | 42460 | 47896 | 5435 |
| T | 5 | MXF12 | 3463 | 8512 | 5048 | T | 3 | F24K9 | 1045 | 6630 | 5584 |
| T | 2 | F3L12 | 10337 | 15413 | 5075 | T | 4 | F7L13 | 39675 | 45336 | 5660 |
| T | 1 | T18A20 | 20393 | 25490 | 5096 | T | 2 | F13B15 | 73787 | 79470 | 5682 |
| T | 4 | FCA8 | 97366 | 102508 | 5141 | T | 5 | MCA23 | 35606 | 41303 | 5696 |
| T | 4 | F1C12 | 29670 | 34842 | 5171 | T | 1 | F7G19 | 26024 | 31724 | 5699 |
| T | 1 | F5I14 | 28596 | 33866 | 5269 | T | 2 | T3P4 | 9772 | 15484 | 5711 |
| T | 1 | T5I8 | 54107 | 59406 | 5298 | T | 4 | F21P8 | 14429 | 20155 | 5725 |
| T | 3 | F10A16 | 20950 | 26275 | 5324 | T | 2 | F9A16 | 48001 | 53761 | 5759 |
| T | 3 | MEC18 | 41240 | 46584 | 5343 | T | 3 | MJG9 | 15239 | 21173 | 5933 |
| T | 1 | T18K17 | 2549 | 8625 | 6075 | T | 5 | MOP9 | 32456 | 39336 | 6879 |
| T | 5 | T6I14 | 2622 | 8712 | 6089 | T | 3 | MJG9 | 5592 | 12590 | 6997 |
| T | 4 | T26M18 | 32292 | 38454 | 6161 | T | 4 | F16A16 | 28632 | 35766 | 7133 |
| T | 3 | MOA2 | 43432 | 49707 | 6274 | T | 3 | T4P3 | 50546 | 57735 | 7188 |
| T | 5 | T22N19 | 26991 | 33288 | 6296 | T | 2 | T13L16 | 40032 | 47293 | 7260 |
| T | 3 | F17J16 | 1154 | 7695 | 6540 | T | 5 | MBK20 | 68642 | 76100 | 7457 |
| T | 3 | MIG10 | 8862 | 15485 | 6622 | T | 1 | F16N3 | 48028 | 55527 | 7498 |
| T | 4 | F21P8 | 20595 | 27271 | 6675 | T | 2 | F5O4 | 48511 | 56016 | 7504 |
| T | 2 | F9O13 | 27447 | 34174 | 6726 | T | 3 | F27H5 | 14627 | 22133 | 7505 |
| T | 1 | T6H22 | 23821 | 30584 | 6762 | T | 1 | F16N3 | 131729 | 139357 | 7627 |
| T | 1 | F13O11 | 77927 | 85586 | 7658 | T | 3 | MFD22 | 14642 | 23475 | 8832 |
| T | 2 | T16I21 | 38253 | 45998 | 7744 | T | 1 | F2J6 | 72103 | 81241 | 9137 |
| T | 2 | F3K23 | 84529 | 92323 | 7793 | T | 5 | T21B4 | 51177 | 60350 | 9172 |
| T | 5 | T1E3 | 37096 | 44995 | 7898 | T | 1 | F16N3 | 93932 | 103220 | 9287 |
| T | 4 | FCA3 | 47214 | 55173 | 7958 | T | 1 | T10P12 | 64488 | 73824 | 9335 |
| T | 2 | F27L4 | 46831 | 54927 | 8095 | T | 1 | T4K22 | 2930 | 12295 | 9364 |
| T | 3 | MOD1 | 949 | 9097 | 8147 | T | 1 | F15D2 | 14519 | 23934 | 9414 |
| T | 2 | F13K3 | 66619 | 74886 | 8266 | T | 2 | T24I21 | 96966 | 106733 | 9766 |
| T | 1 | T23E23 | 27963 | 36285 | 8321 | T | 3 | MIE1 | 1525 | 11378 | 9852 |
| T | 4 | FCA7 | 60406 | 69100 | 8693 | T | 2 | F7B19 | 22149 | 32312 | 10162 |
| T | 2 | F3L12 | 27171 | 37377 | 10205 | T | 5 | MMG4 | 44008 | 57775 | 13766 |
| T | 1 | T3F24 | 36749 | 47227 | 10477 | T | 2 | F23H14 | 28142 | 42196 | 14053 |
| T | 3 | F11C1 | 2484 | 12962 | 10477 | T | 3 | MDC11 | 15093 | 29273 | 14179 |
| T | 3 | MZB10 | 69327 | 79809 | 10481 | T | 1 | T5A14 | 70087 | 84415 | 14327 |
| T | 2 | F27O10 | 43813 | 54909 | 11095 | T | 1 | F5A9 | 2407 | 16742 | 14334 |
| T | 1 | F7A19 | 62905 | 74689 | 11783 | T | 2 | T10J7 | 62796 | 77309 | 14512 |
| T | 1 | F22O13 | 86344 | 98622 | 12277 | T | 1 | F15C21 | 1902 | 16462 | 14559 |
| T | 2 | F16P2 | 81313 | 93591 | 12277 | T | 5 | F5H8 | 32244 | 47464 | 15219 |
| T | 3 | MFE16 | 16468 | 28746 | 12277 | T | 3 | MZN24 | 42367 | 57977 | 15609 |
| T | 5 | F14I23 | 49877 | 63472 | 13594 | T | 1 | F19C24 | 7135 | 23291 | 16155 |
| T | 1 | T4M14 | 3056 | 19913 | 16856 | T | 1 | T17H3 | 15877 | 34956 | 19078 |
| T | 2 | F27C12 | 57871 | 74817 | 16945 | T | 1 | T2E6 | 43730 | 68815 | 25084 |
| T | 1 | T24D18 | 53686 | 71213 | 17526 | T | 4 | F5K24 | 62059 | 94293 | 32233 |
| T | 4 | FCA7 | 69201 | 107786 | 38584 |  |  |  |  |  |  |
