# Supplementary material for: Insertions/Deletions-Associated Nucleotide Polymorphism in Arabidopsis thaliana
Source: Front Plant Sci. 2016 Nov 30;7:1792. doi: 10.3389/fpls.2016.01792 (PMC5127803; doi:10.3389/fpls.2016.01792)
Supplement: Supplementary file 2 [file Table2.DOCX]

**Supplementary Table S2.** Genotyping results of 18 indel loci.

| **RGD1** | Col-0 | ***Ler-0*** | ***Sorbo*** | ***Bur-0*** | ***Ag-0*** | ***Hr-5*** | ***Ct-1*** | ***Kil*** | ***Hs-12*** |
| --- | --- | --- | --- | --- | --- | --- | --- | --- | --- |
| **45.5%** | + | + | - | + | - | + | + | + | - |
|  | ***Mt-0*** | ***Kz-9*** | ***Puz-7*** | ***Lov-1*** | ***Nfc-5*** | ***Rf-4*** | ***Wu-0*** | ***Yo-0*** | ***Rrs-7*** |
|  | + | - | + | + | - | - | - | - | - |
|  | ***Zu-0*** | ***Gr-24*** | ***Lwe*** | Gu-0 | Cvi-0 | Edi-0 | Bla-2 | Ang-0 | Got-7 |
|  | - | + | + | - | - | - | - | - | - |
|  | Kas-0 | Nd-1 | C24 | Pog-0 | Kz-7 | Puz-8 | Lip-0 | Rrs-10 | Lov-5 |
|  | + | + | - | + | - | + | - | - | + |
|  | Ts-1 | Tsu-1 | Up-14 | Tamm-2 | Ms-0 | Ws-0 | Zdr-6 | Sq-8 |  |
|  | + | - | - | - | + | - | + | + |  |
| **RGD2** | Col-0 | L*er*-0 | ***Ang-0*** | ***Fm-15*** | ***Kil*** | ***Kz-7*** | ***Kz-9*** | ***Rf-4*** | ***Lov-1*** |
| **40.9%** | + | + | + | + | + | - | - | + | - |
|  | ***Mt-0*** | ***Nd-1*** | ***Zdr-6*** | ***Rrs-7*** | ***Ws-0*** | ***Wu-0*** | ***Yo-0*** | ***Roc*** | ***Hr-5*** |
|  | - | - | - | - | - | + | + | + | + |
|  | ***Puz-7*** | ***Gr-24*** | ***Lwe*** | ***Zu-0*** | Bur-0 | Ct-1 | Cvi-0 | Edi-0 | C24 |
|  | - | + | - | + | + | - | - | + | + |
|  | Gu-0 | Ms-0 | Kas-0 | Bla-2 | Puz-8 | Nfc-5 | Pog-0 | Lip-0 | Rrs-10 |
|  | - | - | + | + | - | + | + | - | + |
|  | Sorbo | Sq-8 | Ts-1 | Tamm-2 | Tsu-1 | Got-7 | Lov-5 | Up-14 | |
|  | + | + | + | - | + | + | - | + |  |
| **RGD3** | Col-0 | L*er*-0 | ***Ang-0*** | ***Bla-2*** | ***Bur-0*** | ***Kz-7*** | ***Kz-9*** | ***Hr-5*** | ***Gr-24*** |
| **42.5%** | + | - | + | - | + | - | - | - | + |
|  | ***Lip-0*** | ***Ms-0*** | ***Lov-1*** | ***Tamm-2*** | ***Nfc-5*** | ***Rf-4*** | ***Nd-0*** | ***Rrs-7*** | ***Sorbo*** |
|  | + | - | - | - | - | + | - | + | + |
|  | ***Ws-0*** | ***Wu-0*** | ***Mt-0*** | ***Zu-0*** | C24 | Ct-1 | Cvi-0 | Edi-0 | Got-7 |
|  | - | - | - | - | - | + | - | + | + |
|  | Kas-0 | Puz-7 | Puz-8 | Rrs-10 | Yo-0 | Zdr-6 | Gu-0 | Lov-5 | Pog-0 |
|  | + | - | - | + | + | - | + | - | - |
|  | Tsu-1 | Up-14 | Ts-1 | Sq-8 |  |  |  |  |  |
|  | + | - | - | - |  |  |  |  |  |
| **NRD1** | Col-0 | ***Ler-0*** | ***Ab-27*** | ***Tamm-2*** | ***Bla-2*** | ***Lip-0*** | ***Ms-0*** | ***Nfc-5*** | ***Sorbo*** |
| **57.1%** | + | - | + | - | + | - | + | + | - |
|  | ***Cs25*** | ***Kil*** | ***Pog-0*** | ***Puz-7*** | ***Kz-9*** | ***Anh-3*** | ***Ts-5*** | ***Sf-2*** | ***Up-14*** |
|  | - | + | + | - | - | + | - | - | + |
|  | ***Ct-1*** | ***Zu*** | ***Yo-0*** | Bur-0 | Cvi-0 | Nfe6 | La-0 | Rf-4 | Laz1 |
|  | + | - | + | + | + | + | + | + | - |
|  | Ag-0 | Bg-4 | Tsu-1 | Rrs-10 | C24 | Nd-1 | Sq-1 | Ts-1 | Rrs-7 |
|  | - | - | + | + | + | - | - | - | - |
|  | Ef-1 | Fm-15 | Got-7 | Gr-24 | Gu-0 | Kas-1 |  |  |  |
|  | + | + | + | + | + | - |  |  |  |
| **NRD2** | Col-0 | ***Ler-0*** | ***Anh-3*** | ***Tamm-46*** | ***Ab-27*** | ***Rf-4*** | ***Ms-0*** | ***Wu*** | ***Puz-7*** |
| **38.1%** | + | - | - | + | - | - | - | - | + |
|  | ***Est-1*** | ***Yo-0*** | ***C24*** | ***Puz-23*** | ***Kas-1*** | ***Mr-0*** | ***Gu-0*** | ***Got-7*** | Co-0 |
|  | + | - | + | - | + | + | + | + | + |
|  | Oy-0 | Bg-4 | Uod-7 | Rrs-10 | Ts-1 | Puz-8 | Nd-1 | Rip-1 | Ws-0 |
|  | + | - | + | - | - | - | + | - | + |
|  | Ag-0 | Nfc5 | Lov-5 | Pog | Cs25 | Cvi-0 | Ef-1 | Fm-15 | Kz-13 |
|  | - | - | - | - | - | - | - | - | - |
|  | Tsu-1 | Up-14 | Bur-0 | Tamm-2 | Laz1 | Rrs-7 |  |  |  |
|  | - | - | + | + | - | - |  |  |  |
| **NRD3** | Col-0 | ***Ler-0*** | ***Bur-0*** | ***Uod-7*** | ***C24*** | ***Got-7*** | ***Ef-1*** | ***Ms-0*** | ***Lip-0*** |
| **25.4%** | + | - | - | + | + | - | + | + | + |
|  | ***Tsu-0*** | ***Mz-0*** | ***Nfc-5*** | ***Puz-23*** | ***Wu*** | ***Sorbo*** | ***Ws-0*** | ***Up-14*** | Hs-12 |
|  | + | - | + | - | + | - | - | - | - |
|  | Ab-27 | An-1 | Ang-0 | Tamm-2 | Gr-24 | Gu-0 | Rrs-7 | Cs25 | Cvi-0 |
|  | - | - | - | - | - | - | + | - | + |
|  | Edi-0 | Frd-1 | Fst-1 | Tamm-46 | Fst-6 | Ga-0 | Inv1 | Kz-7 | Kz-9 |
|  | - | + | - | - | - | - | - | - | - |
|  | Lov-5 | Lwe | Mr-0 | Rrs-10 | Oy-0 | Pog | Puz-7 | Rf-4 | Roc |
|  | + | - | - | - | - | - | - | - | - |
|  | Sq-1 | Bg-4 | Ts-1 | Dem-9 | Ts-5 | Sf-2 | Tsu-1 | Zu | Gy-0 |
|  | - | - | - | - | - | - | + | - | - |
|  | Yo-0 | Zdr-6 | Var-6 | Dm16 | Nd-1 |  |  |  |  |
|  | - | + | - | - | - |  |  |  |  |
| **NRD4** | Col-0 | ***Ler-0*** | ***Ab-27*** | ***Kas-1*** | ***Kz-13*** | ***Zu-0*** | ***Lov-1*** | ***Tsu-0*** | ***Up-14*** |
| **45.8%** | + | - | - | + | + | + | - | + | - |
|  | C24 | Bur-0 | Rrs-7 | Tamm-2 | Cvi-0 | Got-7 | Gu-0 | Zdr-6 | Ws-0 |
|  | - | - | + | + | - | - | - | + | + |
|  | Lov-5 | Ms-0 | Tsu-1 | Rrs-10 | Sq-1 | Ts-1 |  |  |  |
|  | - | - | + | - | - | + |  |  |  |
| **NRD5** | Col-0 | ***Ler-0*** | ***Ab-27*** | ***Anh-3*** | ***Wu-0*** | ***Ws-0*** | ***Sq-1*** | ***Ms-0*** | ***Est-1*** |
| **37.5%** | + | - | - | + | + | + | - | + | - |
|  | Bur-0 | C24 | Kz-13 | Cvi-0 | Ts-1 | Got-7 | Gu-0 | Zdr-6 | Zu-0 |
|  | - | - | + | - | - | - | - | + | - |
|  | Lov-1 | Lov-5 | Up-14 | Rrs-7 | Tsu-0 | Tsu-1 |  |  |  |
|  | - | - | - | - | + | + |  |  |  |
| **NRD6** | Col-0 | ***Ler-0*** | ***Ab-27*** | ***Lov-1*** | ***C24*** | ***Tsu-0*** | ***Gu-0*** | ***Ws-0*** | ***Kz-13*** |
| **42.9%** | + | - | + | - | + | + | + | - | - |
|  | Bur-0 | Rrs-7 | Rrs-10 | Tamm-2 | Ts-1 | Tsu-1 | Cvi-0 | Wu-0 | Zdr-6 |
|  | - | - | + | + | + | + | - | - | - |
|  | Lov-5 | Got-7 | Zu-0 |  |  |  |  |  |  |
|  | - | - | - |  |  |  |  |  |  |
| **NRD7** | Col-0 | ***Ler-0*** | ***Fst-1*** | ***Tsu-0*** | ***Sq-1*** | ***Gu-0*** | ***Ms-0*** | ***Zdr-6*** | Ab-27 |
| **36.4%** | + | - | + | + | - | - | + | - | + |
|  | Bur-0 | C24 | Cvi-0 | Rrs-10 | Rrs-7 | Got-7 | Tsu-1 | Up-14 | Ws-0 |
|  | - | + | + | + | - | + | + | + | - |
|  | Ts-1 | Zu-0 | Lov-1 | Tamm-2 | |  |  |  |  |
|  | + | + | + | - |  |  |  |  |  |
| **NRD8** | Col-0 | ***Ler-0*** | ***Tsu-0*** | ***Puz-23*** | ***Bur-0*** | ***Mr-0*** | ***Kz-7*** | ***Est-1*** | ***Fst-1*** |
| **31.0%** | + | - | - | + | - | + | + | + | - |
|  | Ab-27 | Anh-3 | Lov-5 | Tamm-2 | Ms-0 | Cvi-0 | Puz-7 | Rf-4 | Got-7 |
|  | + | + | - | - | + | + | + | - | + |
|  | C24 | Ts-1 | Tsu-1 | Rrs-10 | Ws-0 | Zdr-6 | Zu | Kz-13 | Sq-1 |
|  | + | + | - | - | + | + | + | + | + |
|  | Gu-0 | Rrs-7 |  |  |  |  |  |  |  |
|  | + | + |  |  |  |  |  |  |  |
| **NRD9** | Col-0 | ***Ler-0*** | ***Uod-7*** | ***Sorbo*** | ***Ab-7*** | ***Ag-0*** | ***An-1*** | ***Bur-0*** | ***Rip-0*** |
| **19.4%** | + | - | + | + | + | - | + | - | - |
|  | C24 | Anh-3 | Kz-9 | Anh-17 | Got-7 | Cvi-0 | Lov-5 | Ts-1 | Tsu-1 |
|  | + | + | + | + | - | + | + | - | + |
|  | Mr-0 | Pog | Rrs-10 | Tamm-2 | Nd-1 | Oy-0 | Rrs-7 | Ws-0 | Lov-1 |
|  | + | + | + | + | + | + | + | + | + |
|  | Up-14 | Yo-0 | Zdr-6 | Zu |  |  |  |  |  |
|  | + | + | + | + |  |  |  |  |  |
| **NRD10** | Col-0 | ***Ler-0*** | ***Ag-0*** | ***Ef-1*** | ***Got-7*** | ***Gu-0*** | ***Hr-5*** | ***Yo-0*** | ***Zdr-6*** |
| **21.2%** | + | - | - | + | - | - | + | + | + |
|  | Ab-27 | Ab-7 | Anh-17 | Rrs-10 | Bur-0 | C24 | Est-1 | Frd-1 | Dem-9 |
|  | - | - | - | + | - | - | - | - | - |
|  | Gr-24 | Kil | Edi-0 | Puz-23 | An-1 | Mr-0 | Ms-0 | Mz-0 | Nd-1 |
|  | - | - | - | - | + | - | - | - | - |
|  | Lov-1 | Tsu-0 | Rf-4 | Rip-1 | Tsu-1 | Sf-2 | Up-14 | Var-6 | Wu |
|  | - | - | - | - | - | - | - | + | - |
|  | Ws-0 | Zu |  |  |  |  |  |  |  |
|  | - | - |  |  |  |  |  |  |  |
| **NRD11** | Col-0 | ***Ler-0*** | ***Anh-3*** | ***Rrs-10*** | ***Est-1*** | ***Mr-0*** | ***Ms-0*** | ***Rf-4*** | ***Oy-0*** |
| **16.7%** | + | - | - | + | - | + | - | + | + |
|  | Ab-27 | Ab-7 | Bur-0 | Rrs-10 | C24 | Cvi-0 | Got-7 | Gr-24 | Gu-0 |
|  | + | + | + | + | + | + | + | + | + |
|  | Ef-1 | Fst-1 | Rrs-7 | Sq-1 | Ts-1 | Lov-1 | Tsu-0 | Tsu-1 | Up-14 |
|  | + | + | + | + | + | + | + | + | + |
|  | Ws-0 | Zdr-6 | Zu |  |  |  |  |  |  |
|  | + | - | + |  |  |  |  |  |  |
| **NRD12** | Col-0 | ***Ler-0*** | ***Bur-0*** | ***Gu-0*** | ***Kas-1*** | ***Kz-13*** | ***Kz-9*** | ***Sq-1*** | ***Up-14*** |
| **17.9%** | + | - | + | + | - | - | - | + | + |
|  | Ab-27 | Ag-0 | Ang-0 | Anh-3 | Bg-4 | An-1 | C24 | Dm16 | Ef-1 |
|  | + | + | + | + | - | + | + | + | + |
|  | Frd-1 | Fst-6 | Got-7 | Kz-1 | Gy-0 | Hs-12 | Inv1 | La-0 | Lip-0 |
|  | + | + | + | - | - | + | + | - | + |
|  | Mt | Nd-1 | Oy-0 | Tamm-46 | Puz-7 | Rf-4 | Rip-1 | Roc | Rrs-10 |
|  | + | + | + | + | + | + | + | - | + |
|  | Sf-2 | Sorbo | Lov-5 | Tamm-2 | Pog | Bla-2 | Ts-1 | Tsu-0 | Tsu-1 |
|  | + | + | + | + | + | + | + | + | + |
|  | Mr-0 | Var-6 | Ws-0 | Uod-7 | Zdr-6 | Zu | Cvi-0 | Kz-7 | Ms-0 |
|  | + | + | + | + | + | + | + | - | + |
|  | Rrs-7 | Wu |  |  |  |  |  |  |  |
|  | + | + |  |  |  |  |  |  |  |
| **NRD13** | Col-0 | ***Ler-0*** | ***Bur-0*** | ***Kz-13*** | ***Sq-1*** | ***Tsu-0*** | ***Ms-0*** | ***Zdr-6*** | ***Up-14*** |
| **33.3%** | + | -- | - | + | + | + | -- | + | - |
|  | Ab-27 | C24 | Cvi-0 | Rrs-10 | Gu-0 | Lov-1 | Rrs-7 | Ts-1 | Tsu-1 |
|  | - | - | - | -- | + | + | -- | -- | -- |
|  | Tsu-0 | Ws-0 | Wu-0 | Zu-0 |  |  |  |  |  |
|  | + | - | - | - |  |  |  |  |  |
| **NRD14** | Col-0 | ***Ler-0*** | ***Lov-1*** | ***Kas-1*** | ***Ms-0*** | ***Sq-1*** | ***Ws-0*** | ***Zdr-6*** | Ab-27 |
| **33.3%** | + | - | - | + | + | + | - | - | + |
|  | C24 | Got-7 | Bur-0 | Rrs-10 | Cvi-0 | Zu-0 | Gu-0 | Up-14 | Kz-13 |
|  | - | - | + | + | + | + | + | + | + |
|  | Ts-1 | Tsu-0 | Tsu-1 | Tamm-2 | Rrs-7 | Wu-0 |  |  |  |
|  | + | - | - | + | + | + |  |  |  |
| **NRD15** | Col-0 | ***Ler-0*** | ***Dem-9*** | ***Anh-17*** | ***Ab-7*** | ***Lov-1*** | ***Ren-1*** | ***Ws-0*** | ***Sorbo*** |
| **37.0%** | + | - | - | - | - | + | + | + | + |
|  | Ag-0 | An-1 | Rrs-10 | Puz-23 | C24 | Got-7 | Cvi-0 | Kz-9 | Bur-0 |
|  | + | + | - | + | + | - | + | - | + |
|  | Ga-0 | Rrs-7 | Pog | Tamm-2 | Ts-1 | Tsu-1 | Up-14 | Lip | Rf-4 |
|  | - | + | + | + | + | + | - | + | - |

“+” means indel presence. “-” means indel absence and “--” means larger indel absence in two-indel loci. The italic accessions are those sequenced in this study. The frequency of each indel polymorphism is listed below the locus name.
