# Supplementary material for: Insertions/Deletions-Associated Nucleotide Polymorphism in Arabidopsis thaliana
Source: Front Plant Sci. 2016 Nov 30;7:1792. doi: 10.3389/fpls.2016.01792 (PMC5127803; doi:10.3389/fpls.2016.01792)
Supplement: Supplementary file 3 [file Table3.DOCX]

**Supplementary Table S3.** Statistics for 15 randomly-selected indel loci in the sequenced regions.

| Type | Locus | Indel size (bp) | Position to DJ | Sequenced accessions | Length (bp) | Fixed sites | | Nucleotide variation | | | Tajima's D |
| --- | --- | --- | --- | --- | --- | --- | --- | --- | --- | --- | --- |
|  |  |  |  |  |  | S_N_ | indel | π_t_ | π_fixed_ | d_xy_ |  |
| NRD | 1 | 4524 | JR' | 21 | 785 | 4 | 0 | 0.0075 | 0.0027 | 0.0052 | 0.61 |
|  |  |  | JR | 21 | 600 | 4 | 0 | 0.0092 | 0.0035 | 0.0067 | 0.84 |
|  |  |  | 3'-0.3kb | 21 | 170 | 0 | 0 | 0.0021 | 0.0000 | 0.0000 | -0.84 |
|  | 2 | 5584 | JR' | 17 | 2442 | 14 | 3 | 0.0081 | 0.0027 | 0.0057 | 1.01 |
|  |  |  | 5'-0.3kb | 17 | 1102 | 0 | 0 | 0.0026 | 0.0000 | 0.0000 | -0.42 |
|  |  |  | JR | 17 | 600 | 14 | 3 | 0.0197 | 0.0109 | 0.0233 | 1.77 |
|  |  |  | 3'-0.3kb | 17 | 740 | 0 | 0 | 0.0068 | 0.0000 | 0.0000 | 0.53 |
|  | 3 | 1076 | JR' | 17 | 2651 | 27 | 1 | 0.0201 | 0.0058 | 0.0109 | 1.10 |
|  |  |  | 5'-0.3kb | 17 | 1099 | 1 | 1 | 0.0143 | 0.0005 | 0.0010 | 0.22 |
|  |  |  | JR | 17 | 600 | 25 | 0 | 0.0287 | 0.0261 | 0.0417 | 2.51** |
|  |  |  | 3'-0.3kb | 17 | 952 | 1 | 0 | 0.0216 | 0.0006 | 0.0011 | 0.95 |
|  | 4 | 807 | JR' | 9 | 2536 | 31 | 11 | 0.0207 | 0.0071 | 0.0127 | 1.39 |
|  |  |  | 5'-0.3kb | 9 | 1198 | 4 | 1 | 0.0268 | 0.0020 | 0.0035 | 0.86 |
|  |  |  | JR | 9 | 600 | 25 | 8 | 0.0268 | 0.0247 | 0.0417 | 2.31** |
|  |  |  | 3'-0.3kb | 9 | 738 | 2 | 2 | 0.0066 | 0.0015 | 0.0027 | 2.12* |
|  | 5 | 404 | JR' | 9 | 1926 | 11 | 5 | 0.0128 | 0.0036 | 0.0065 | -0.01 |
|  |  |  | 5'-0.3kb | 9 | 618 | 0 | 0 | 0.0113 | 0.0000 | 0.0000 | -1.35 |
|  |  |  | JR | 9 | 600 | 4 | 0 | 0.0123 | 0.0038 | 0.0069 | 1.03 |
|  |  |  | 3'-0.3kb | 9 | 708 | 7 | 5 | 0.0150 | 0.0077 | 0.0139 | 1.01 |
|  | 6 | 101 | JR' | 9 | 2142 | 30 | 4 | 0.0091 | 0.0079 | 0.0141 | 1.59 |
|  |  |  | 5'-0.3kb | 9 | 912 | 22 | 3 | 0.0144 | 0.0136 | 0.0250 | 2.00* |
|  |  |  | JR | 9 | 600 | 6 | 0 | 0.0067 | 0.0056 | 0.0100 | 1.71 |
|  |  |  | 3'-0.3kb | 9 | 630 | 2 | 1 | 0.0038 | 0.0018 | 0.0032 | -0.32 |
|  | 7 | 578 | JR' | 8 | 2172 | 5 | 1 | 0.0098 | 0.0013 | 0.0023 | -0.15 |
|  |  |  | 5'-0.3kb | 8 | 737 | 3 | 1 | 0.0102 | 0.0024 | 0.0041 | 0.31 |
|  |  |  | JR | 8 | 600 | 0 | 0 | 0.0078 | 0.0000 | 0.0000 | 0.47 |
|  |  |  | 3'-0.3kb | 8 | 835 | 2 | 0 | 0.0110 | 0.0014 | 0.0025 | -0.72 |
|  | 8 | 121 | JR' | 9 | 2099 | 3 | 0 | 0.0096 | 0.0008 | 0.0015 | -1.50 |
|  |  |  | 5'-0.3kb | 9 | 682 | 0 | 0 | 0.0046 | 0.0000 | 0.0000 | -1.52 |
|  |  |  | JR | 9 | 600 | 1 | 0 | 0.0138 | 0.0009 | 0.0017 | -1.80* |
|  |  |  | 3'-0.3kb | 9 | 817 | 2 | 0 | 0.0107 | 0.0015 | 0.0027 | -0.97 |
|  | 9 | 135 | JR' | 9 | 2681 | 34 | 6 | 0.0159 | 0.0072 | 0.0130 | 0.32 |
|  |  |  | 5'-0.3kb | 9 | 843 | 0 | 0 | 0.0065 | 0.0000 | 0.0000 | -0.95 |
|  |  |  | JR | 9 | 600 | 15 | 2 | 0.0190 | 0.0140 | 0.0252 | 1.65 |
|  |  |  | 3'-0.3kb | 9 | 1238 | 20 | 4 | 0.0208 | 0.0093 | 0.0168 | 0.18 |
|  | 10 | 1001 | JR' | 9 | 1949 | 2 | 1 | 0.0045 | 0.0006 | 0.0010 | -1.05 |
|  |  |  | 5'-0.3kb | 9 | 676 | 1 | 0 | 0.0047 | 0.0008 | 0.0015 | -0.72 |
|  |  |  | JR | 9 | 600 | 1 | 1 | 0.0042 | 0.0009 | 0.0017 | -1.13 |
|  |  |  | 3'-0.3kb | 9 | 673 | 0 | 0 | 0.0046 | 0.0000 | 0.0000 | -1.09 |
|  | 11 | 1397 | JR' | 9 | 2029 | 9 | 5 | 0.0141 | 0.0026 | 0.0046 | 0.01 |
|  |  |  | 5'-0.3kb | 9 | 727 | 0 | 0 | 0.0162 | 0.0000 | 0.0000 | -0.14 |
|  |  |  | JR | 9 | 600 | 2 | 3 | 0.0057 | 0.0019 | 0.0034 | 0.60 |
|  |  |  | 3'-0.3kb | 9 | 702 | 7 | 2 | 0.0190 | 0.0057 | 0.0103 | 0.00 |
|  | 12 | 304 | JR' | 9 | 880 | 8 | 0 | 0.0127 | 0.0051 | 0.0091 | 0.03 |
|  | 13 | 238/501 | JR' | 9 | 960 | / | / | 0.0122 | / | / | 0.07 |
|  | 14 | 345/340 | JR' | 8 | 2308 | / | / | 0.0121 | / | / | -0.43 |
|  | 15 | 205/335 | JR' | 9 | 2194 | / | / | 0.0042 | / | / | -0.24 |

DJ, JR，JR’ and SN represent deletion junction, junction region, whole region and the nucleotide substitution sites, respectively. *, P < 0.05; **, P < 0.01
