# Supplementary material for: Insertions/Deletions-Associated Nucleotide Polymorphism in Arabidopsis thaliana
Source: Front Plant Sci. 2016 Nov 30;7:1792. doi: 10.3389/fpls.2016.01792 (PMC5127803; doi:10.3389/fpls.2016.01792)
Supplement: Supplementary file 4 [file Table4.DOCX]

**Supplementary Table S4.** Statistics of dimorphic and non-dimorphic loci in Nordborg (Nordborg et al. 2005) data. π_t_ and π_fixed_ are defined in Methods.

| Locus |  | All | Coding | Non-coding |
| --- | --- | --- | --- | --- |
| Dimorphic  loci | Number | 307 | / | / |
|  | Frequency | 27.2% | / | / |
|  | Length | 169032 | 75460 | 93572 |
|  |  | 27.2% | 26.2% | 28.1% |
|  | Fixed substitutions | 3086 | 994 | 2092 |
|  |  | 17.2% | 16.6% | 17.4% |
|  | π_fixed xy_ | 0.0073 | 0.0054 | 0.0088 |
|  |  | 43.0% | 43.9% | 42.6% |
|  | π_fixed xy_/π_t1_ | 67.6% | 67.4% | 67.7% |
|  | π_t1_ | 0.0107 | 0.0080 | 0.0129 |
|  |  | 63.6% | 65.2% | 62.9% |
| The other loci | Number | 823 | / | / |
|  | Frequency | 72.8% | / | / |
|  | Length | 451640 | 212397 | 239243 |
|  |  | 72.8% | 73.8% | 71.9% |
|  | π_t2_ | 0.0023 | 0.0015 | 0.0030 |
|  |  | 36.4% | 34.8% | 37.1% |
| Total | Number | 1130 | 199 | 296 |
|  | SNP sites | 17974 | 5973 | 12001 |
|  | Length | 620672 | 287857 | 332815 |
|  | π_t_ | 0.0046 | 0.0032 | 0.0058 |
