# Supplementary material for: Insertions/Deletions-Associated Nucleotide Polymorphism in Arabidopsis thaliana
Source: Front Plant Sci. 2016 Nov 30;7:1792. doi: 10.3389/fpls.2016.01792 (PMC5127803; doi:10.3389/fpls.2016.01792)
Supplement: Supplementary file 6 [file Image1.PDF]

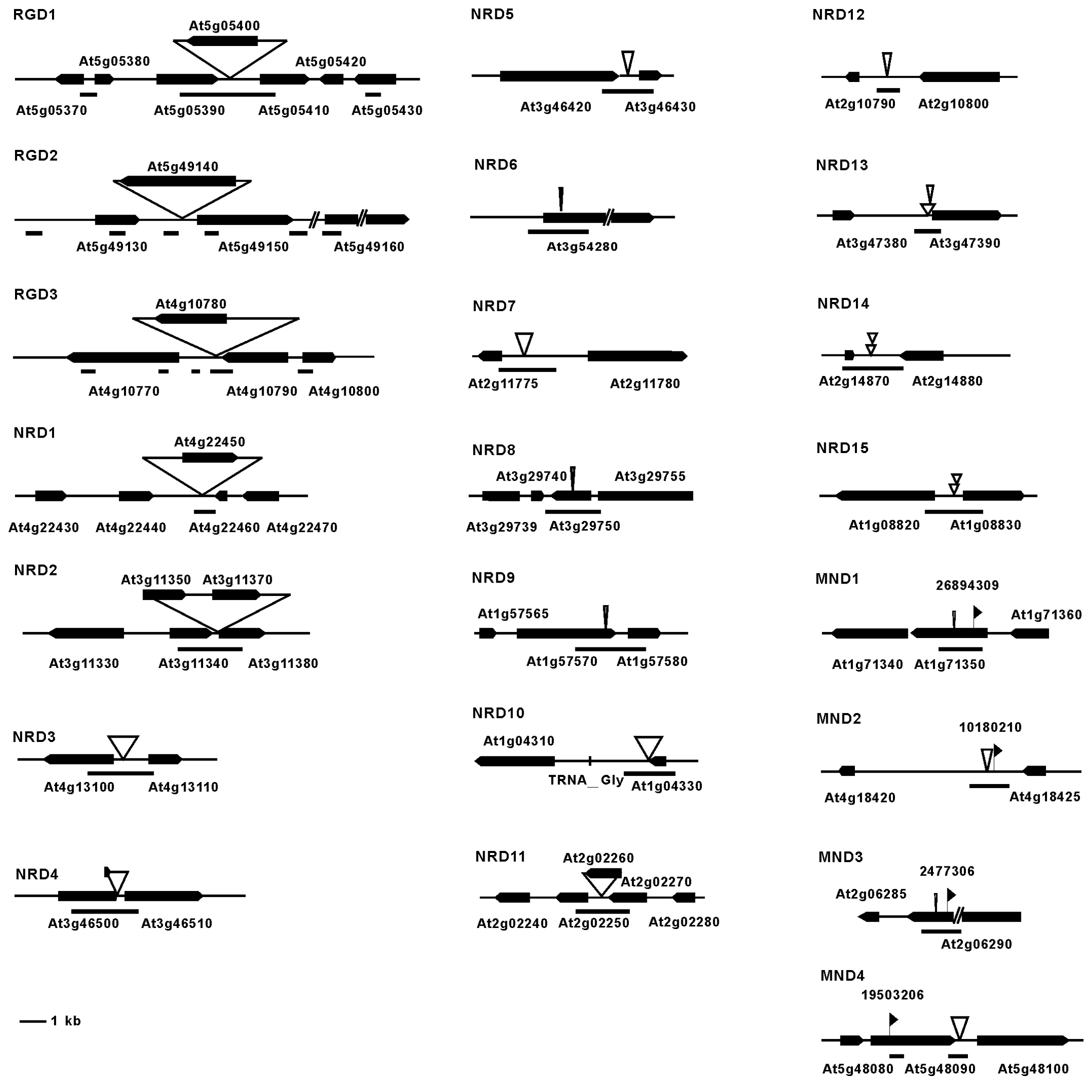

Supplementary Figure S1. Loci surveyed in this study. The indels and their sizes are displayed as hollowed triangles, and genes are displayed as black boxes. The black lines below the gene structures represent sequenced fragments in this study. Two indels at loci NRD13, 14 and 15 represent different patterns of two deletion haplotypes. MND1–4 are four dimorphic loci (indicated by the numbers above the thin arrows) randomly selected from the Nordborg dataset (http://walnut.usc.edu /2010). The thin arrows depict the starting sequencing points in selected loci and hollowed triangles were identified indels around these loci.
