# Supplementary material for: Insertions/Deletions-Associated Nucleotide Polymorphism in Arabidopsis thaliana
Source: Front Plant Sci. 2016 Nov 30;7:1792. doi: 10.3389/fpls.2016.01792 (PMC5127803; doi:10.3389/fpls.2016.01792)
Supplement: Supplementary file 7 [file Image2.PDF]

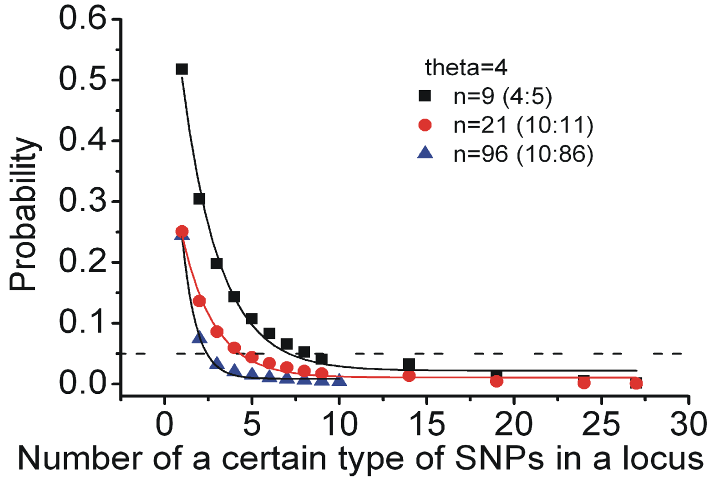

Supplementary Figure S2. Results of coalescent simulations of the probability of a 600bp locus containing a certain numbers of linked SNPs. The simulation is based on a neutral model with constant population size, no recombination, panmixis, and an infinite sites. We repeated the simulation 10000 times, using software developed by Hudson (2002). The mutation rate (θ) is 4 [=S/1215/ $\sum_{j=1}^{n-1}\frac{1}{j}$ /580*600; S is the sum of SNPs and indels obtained from the Nordborg dataset (Nordborg et al. 2005), the sequence length is set as 600 base pairs, the same length as the junction regions in the sequenced 18 indel loci]. The black squares represent n=9 accessions, in which the minor haplotype (or indel) frequency is 4/9; the red cycles represent n=21 accessions with a minor haplotype frequency of 10/21; the blue triangles represent n=96 accessions with a minor haplotype frequency of 10/96.

Notably, $P_{\text{linked SNPs}\geq 3}$ (the probability of a locus having more than 3 mutually linked SNPs) is <0.05 in 96 accessions (the indel stratifies the accessions into 10:86), demonstrating that the dimorphic loci meeting the criteria defined the text, are not random. It also shows that the increase of sequenced accessions does not significantly enhance the power to claim a dimorphism.
