# Supplementary material for: Insertions/Deletions-Associated Nucleotide Polymorphism in Arabidopsis thaliana
Source: Front Plant Sci. 2016 Nov 30;7:1792. doi: 10.3389/fpls.2016.01792 (PMC5127803; doi:10.3389/fpls.2016.01792)
Supplement: Supplementary file 8 [file Image3.PDF]

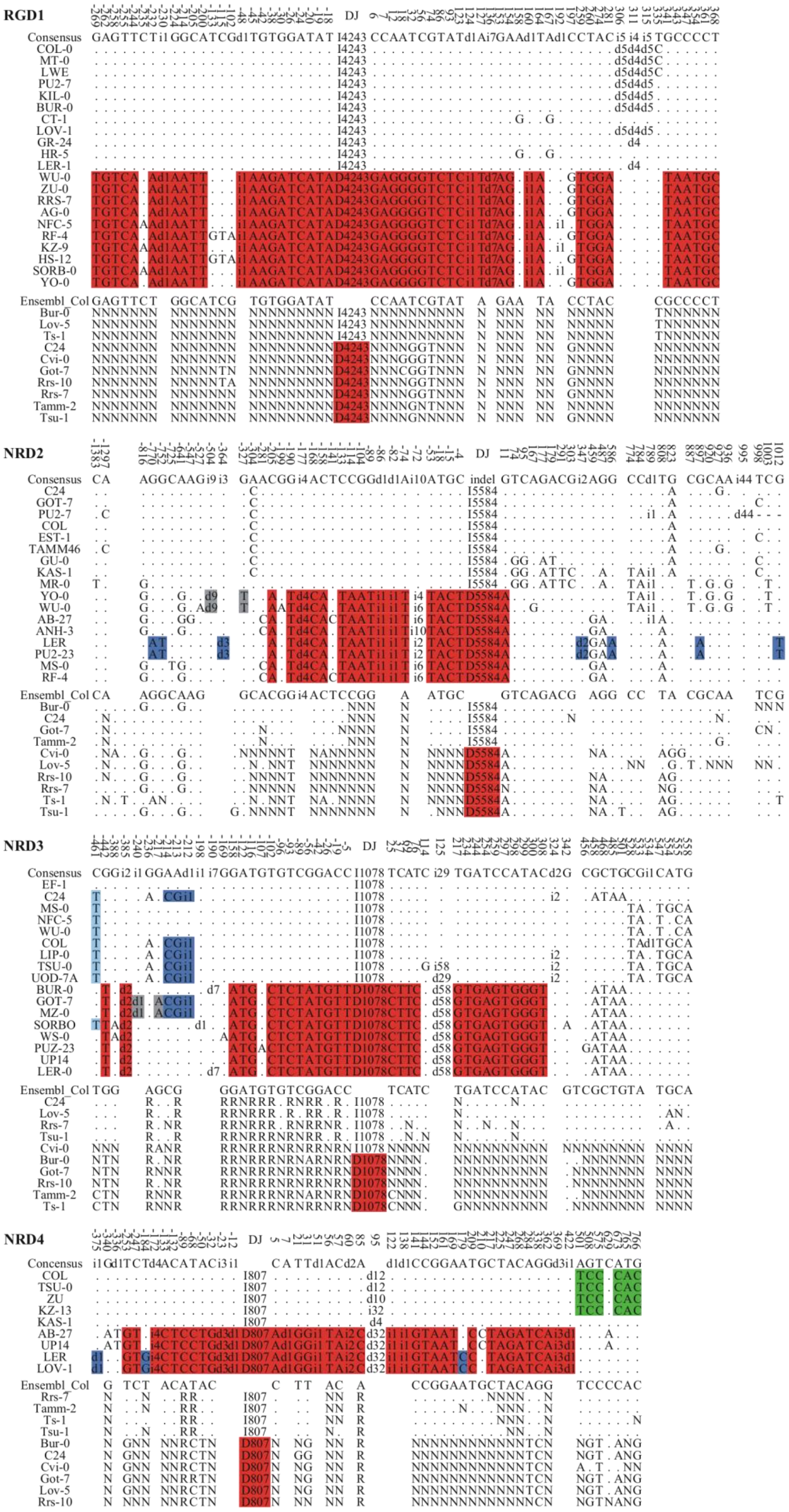
RGD1
NRD2
NRD3
NRD4

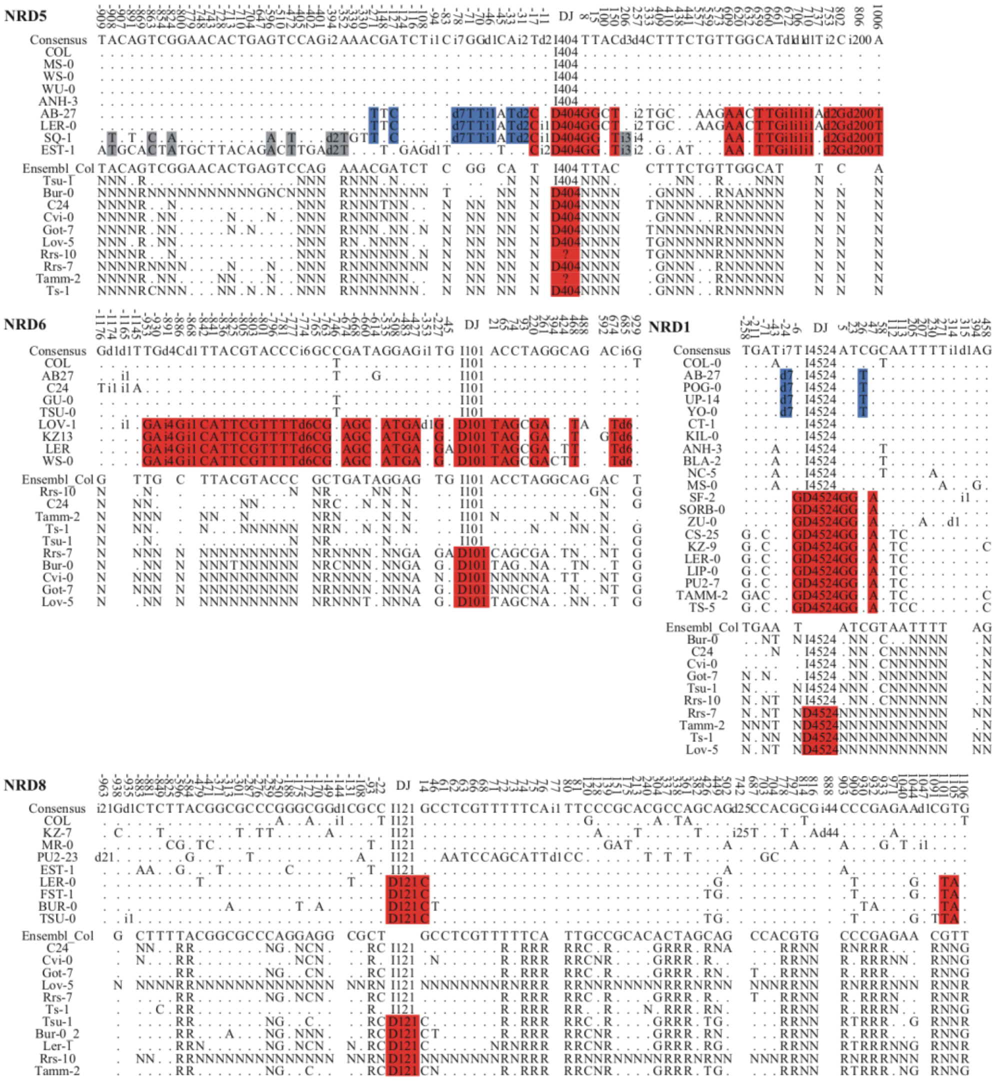

Supplementary Figure S3. Indel presence/absence genotyping results and SNP calls of the 10 additional accessions from Perlegen data (Clark et al, 2007)
