# Supplementary material for: Insertions/Deletions-Associated Nucleotide Polymorphism in Arabidopsis thaliana
Source: Front Plant Sci. 2016 Nov 30;7:1792. doi: 10.3389/fpls.2016.01792 (PMC5127803; doi:10.3389/fpls.2016.01792)
Supplement: Supplementary file 9 [file Image4.PDF]

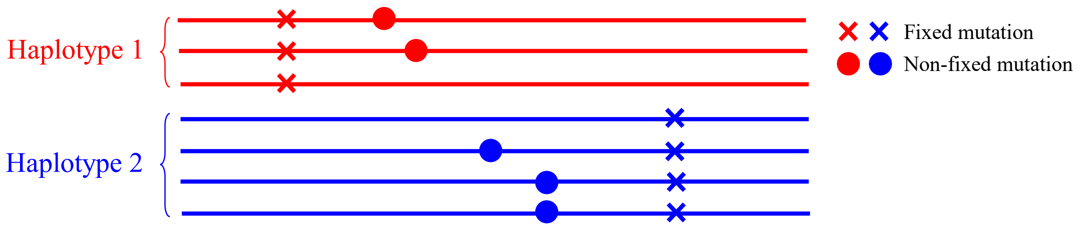

Supplementary Figure S4. Dimorphic diversity (d_{xy}) analysis. The nucleotide diversity in a population is usually estimated by (Nei and Miller, 1990):

$$\pi = 2\sum_{i<j}^{n} d_{ij}/[n(n-1)] \qquad (1)$$

where d_{ij} is an estimate of the number of nucleotide substitutions per site between DNA sequences i and j and n is the number of DNA sequences examined. At a dimorphic locus (haplotype 1 with red color and haplotype 2 with blue color), the nucleotide diversity (Nei, 1987), can be divided into three parts: the contribution by mutations within haplotypes (π_h), the fixed substitutions ($\pi_{fixedxy}$) and the non-fixed substitutions ($\pi_{non\text{-}fixedxy}$) between haplotypes:

$$\pi = \frac{n_1(n_1-1)}{n(n-1)} \times \pi_x + \frac{n_2(n_2-1)}{n(n-1)} \times \pi_y + \frac{2n_1n_2}{n(n-1)} \times D_{xy} \qquad (2)$$

Where n_1 is the number of DNA sequences of haplotype 1, and n_2 is the number of DNA sequences of haplotype 2. $\pi_h = \pi_{h1} + \pi_{h2} = \frac{n_1(n_1-1)}{n(n-1)} \times \pi_x + \frac{n_2(n_2-1)}{n(n-1)} \times \pi_y$, $\frac{2n_1n_2}{n(n-1)} \times D_{xy}$ can be divided into two parts:

$$\frac{2n_1n_2}{n(n-1)} \times D_{xy} = \pi_{fixedxy} + \pi_{non-fixedxy} \qquad (3)$$

Where $\pi_{fixedxy}$ is the mean pairwise nucleotide differences contributed the fixed substitutions (marked as cross) by between two haplotypes and $\pi_{non\text{-}fixedxy}$ is the mean pairwise nucleotide differences contributed the fixed substitutions (marked as circle) by between two haplotypes. When calculating $\pi_{fixedxy}$, the non-fixed substitutions should be ignored. Thus, the nucleotide diversity will equal $\pi_{fixedxy}$. If there was m fixed substitutions between two haplotypes and the length of DNA sequences was N-bp, we would get this based on formula (1): (4)

$$\pi_{fixedxy} = \pi_{without-nonfixed-substitutions} = 2/[n(n-1)] \times m/N \times n_1 \times n_2$$

Here, we defined $d_{xy} = \mathrm{m/N}$, thus,

$$\pi_{fixedxy} = \frac{2n_1n_2}{n(n-1)} d_{xy} \qquad (5)$$

References

Nei, M. (1987). *Molecular evolutionary genetics*. Columbia university press.

Nei, M., and Miller, J. C. (1990). A Simple Method for Estimating Average Number of Nucleotide Substitutions within and between Populations from Restriction Data. *Genetics* 125, 873–879.
