# Supplementary material for: Insertions/Deletions-Associated Nucleotide Polymorphism in Arabidopsis thaliana
Source: Front Plant Sci. 2016 Nov 30;7:1792. doi: 10.3389/fpls.2016.01792 (PMC5127803; doi:10.3389/fpls.2016.01792)
Supplement: Supplementary file 10 [file Image5.PDF]

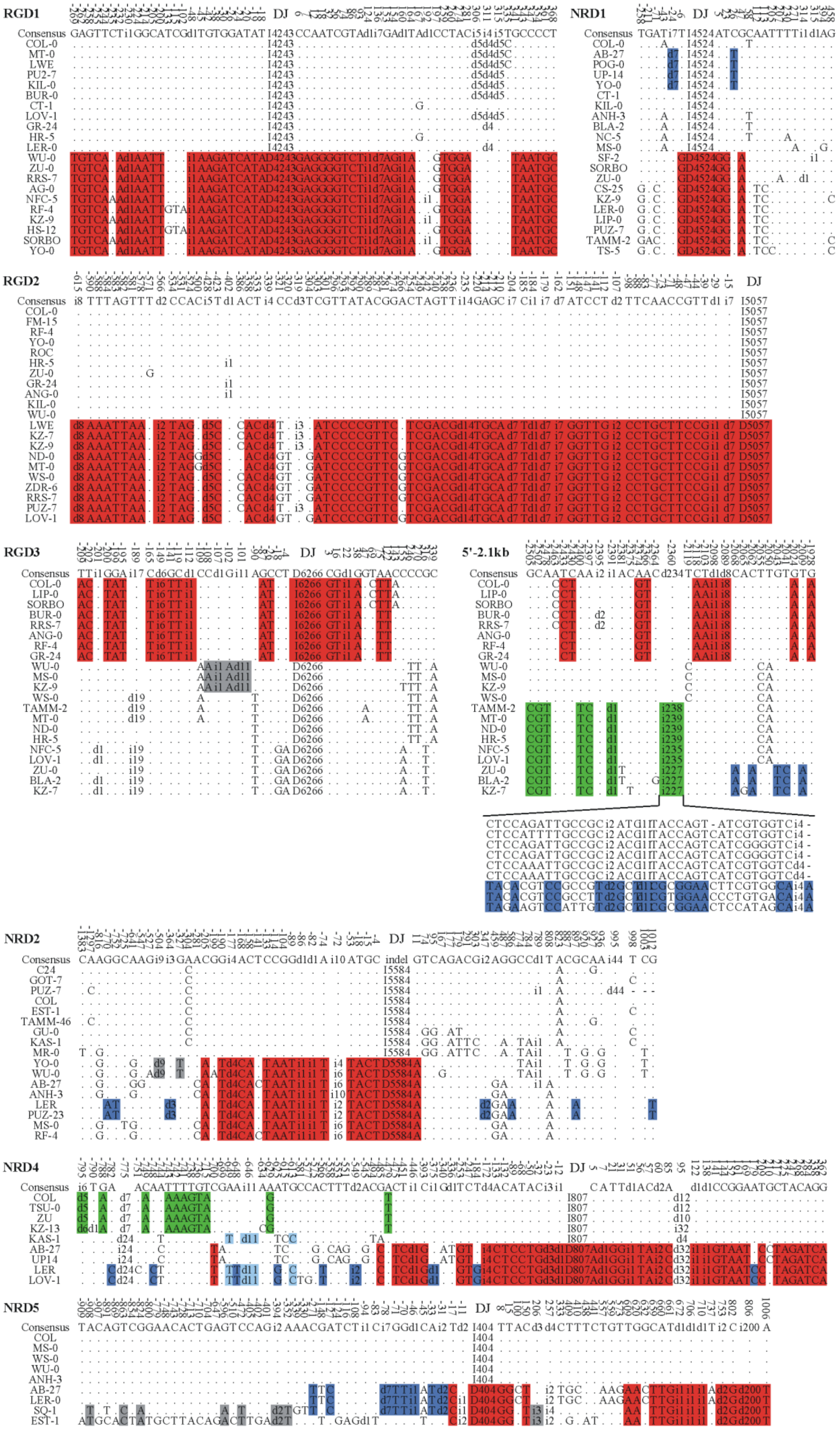
RGD1
Consensus
COL-0
MT-0
LWE
PU2-7
KIL-0
BUR-0
CT-1
LOV-1
GR-24
HR-5
LER-0
WU-0
ZU-0
RRS-7
AG-0
NFC-5
RF-4
KZ-9
HS-12
SORBO
YO-0
NRD1
Consensus
COL-0
AB-27
POG-0
UP-14
YO-0
CT-1
KIL-0
ANH-3
BLA-2
NC-5
MS-0
SF-2
SORBO
ZU-0
CS-25
KZ-9
LER-0
LIP-0
PUZ-7
TAMM-2
TS-5
RGD2
Consensus
COL-0
FM-15
RF-4
YO-0
ROC
HR-5
ZU-0
GR-24
ANG-0
KIL-0
WU-0
LWE
KZ-7
KZ-9
ND-0
MT-0
WS-0
ZDR-6
RRS-7
PUZ-7
LOV-1
RGD3
Consensus
COL-0
LIP-0
SORBO
BUR-0
RRS-7
ANG-0
RF-4
GR-24
WU-0
MS-0
KZ-9
WS-0
TAMM-2
MT-0
ND-0
HR-5
NFC-5
LOV-1
ZU-0
BLA-2
KZ-7
5'-2.1kb
Consensus
COL-0
LIP-0
SORBO
BUR-0
RRS-7
ANG-0
RF-4
GR-24
WU-0
MS-0
KZ-9
WS-0
TAMM-2
MT-0
ND-0
HR-5
NFC-5
LOV-1
ZU-0
BLA-2
KZ-7
NRD2
Consensus
C24
GOT-7
PUZ-7
COL
EST-1
TAMM-46
GU-0
KAS-1
MR-0
YO-0
WU-0
AB-27
ANH-3
LER
PUZ-23
MS-0
RF-4
NRD4
Consensus
COL
TSU-0
ZU
KZ-13
KAS-1
AB-27
UP14
LER
LOV-1
NRD5
Consensus
COL
MS-0
WS-0
WU-0
ANH-3
AB-27
LER-0
SQ-1
EST-1
DJ

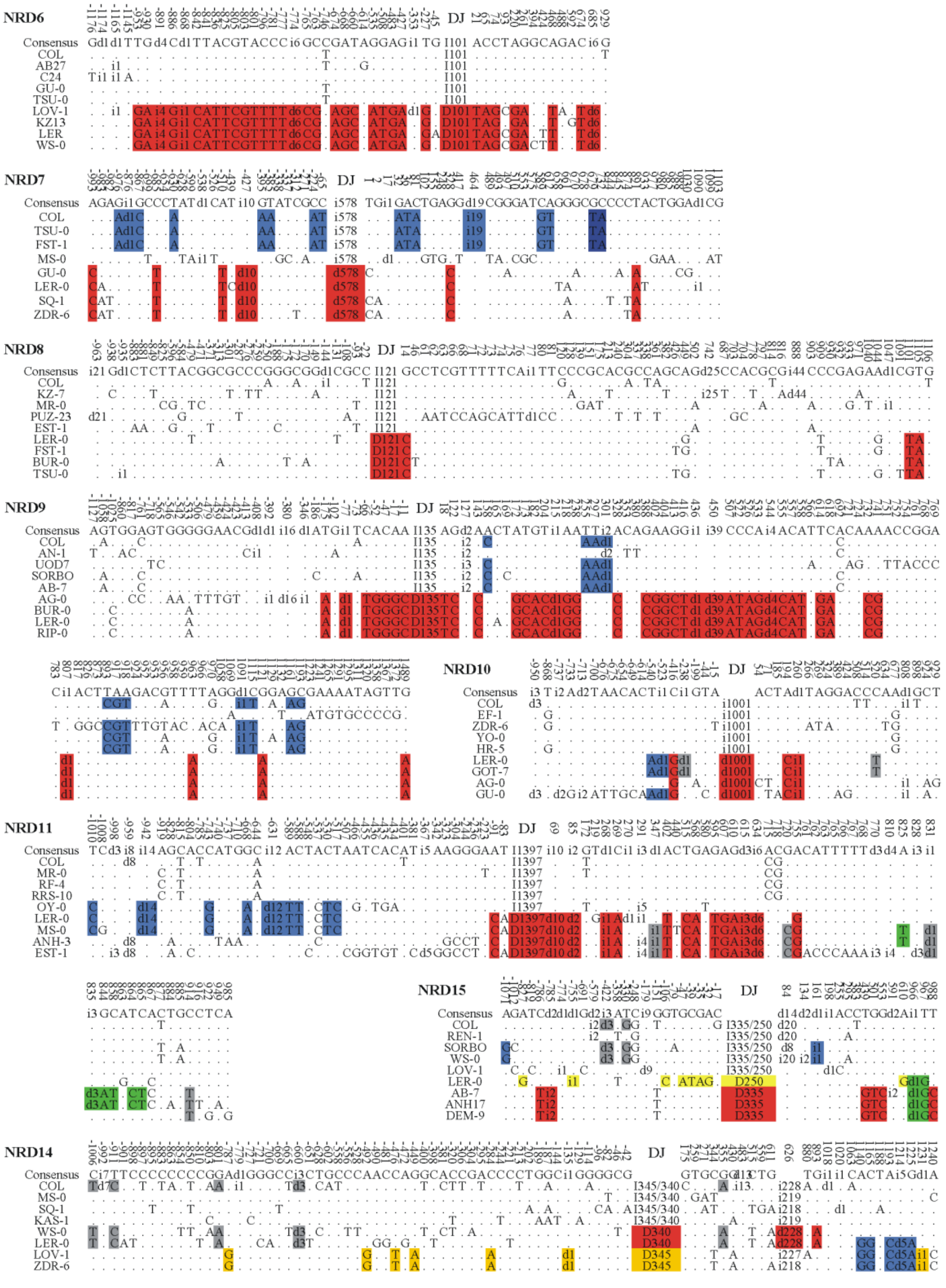

Supplementary Figure S5. Polymorphism in locus RGD1–3 and NRD1–15. The number represents the relative position at each locus to the deletion junction (DJ). The i345 or d340 designation represents either a 345bp insertion or a 340bp deletion. The colored mutations are dimorphic sites and the different-colored sites at each locus represent different patterns of dimorphisms. The red-colored (and yellow-colored at locus M) sites correspond to the major indel polymorphisms and the other colors corresponding to the other indel patterns.
