# Supplementary material for: Insertions/Deletions-Associated Nucleotide Polymorphism in Arabidopsis thaliana
Source: Front Plant Sci. 2016 Nov 30;7:1792. doi: 10.3389/fpls.2016.01792 (PMC5127803; doi:10.3389/fpls.2016.01792)
Supplement: Supplementary file 11 [file Image6.PDF]

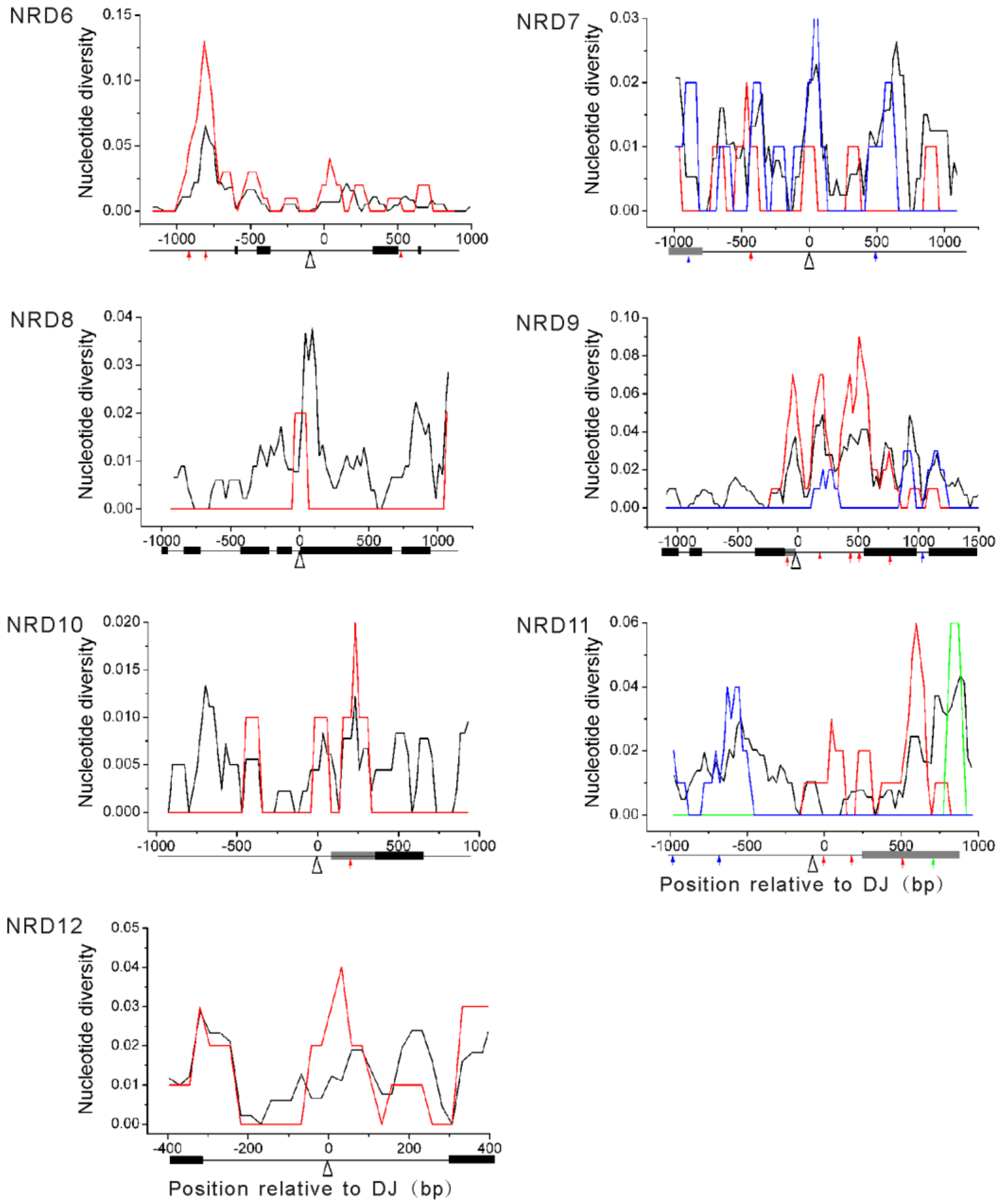

Supplementary Figure S6. Sliding windows of the nucleotide diversity (black line) and the dimorphic diversity (d_{xy}, red and other colored line) at locus NRD6–12. The red line represents the corresponding d_{xy} with the selected indel (the open triangle), and the other colored lines represent dimorphic patterns corresponding to the other indel polymorphisms. Selected indels are positioned at 0. Black boxes represent coding regions.
