# Supplementary material for: Insertions/Deletions-Associated Nucleotide Polymorphism in Arabidopsis thaliana
Source: Front Plant Sci. 2016 Nov 30;7:1792. doi: 10.3389/fpls.2016.01792 (PMC5127803; doi:10.3389/fpls.2016.01792)
Supplement: Supplementary file 12 [file Image7.PDF]

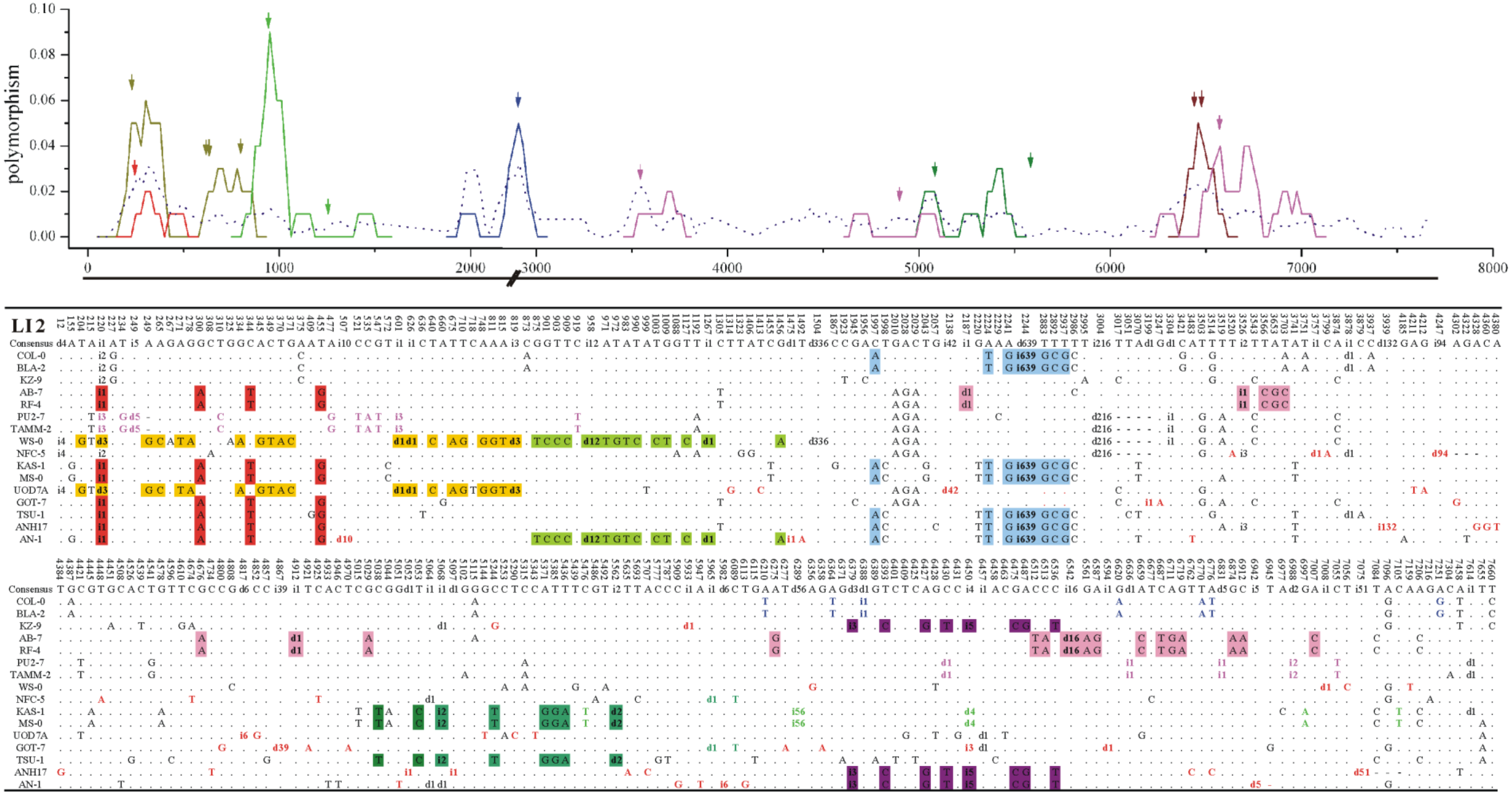

polymorphism
0.10
0.08
0.06
0.04
0.02
0.00
0
1000
2000
3000
4000
5000
6000
7000
8000
LI2
Consensus
COL-0
BLA-2
KZ-9
AB-7
RF-4
PU2-7
TAMM-2
WS-0
NFC-5
KAS-1
MS-0
UOD7A
GOT-7
TSU-1
ANH17
AN-1

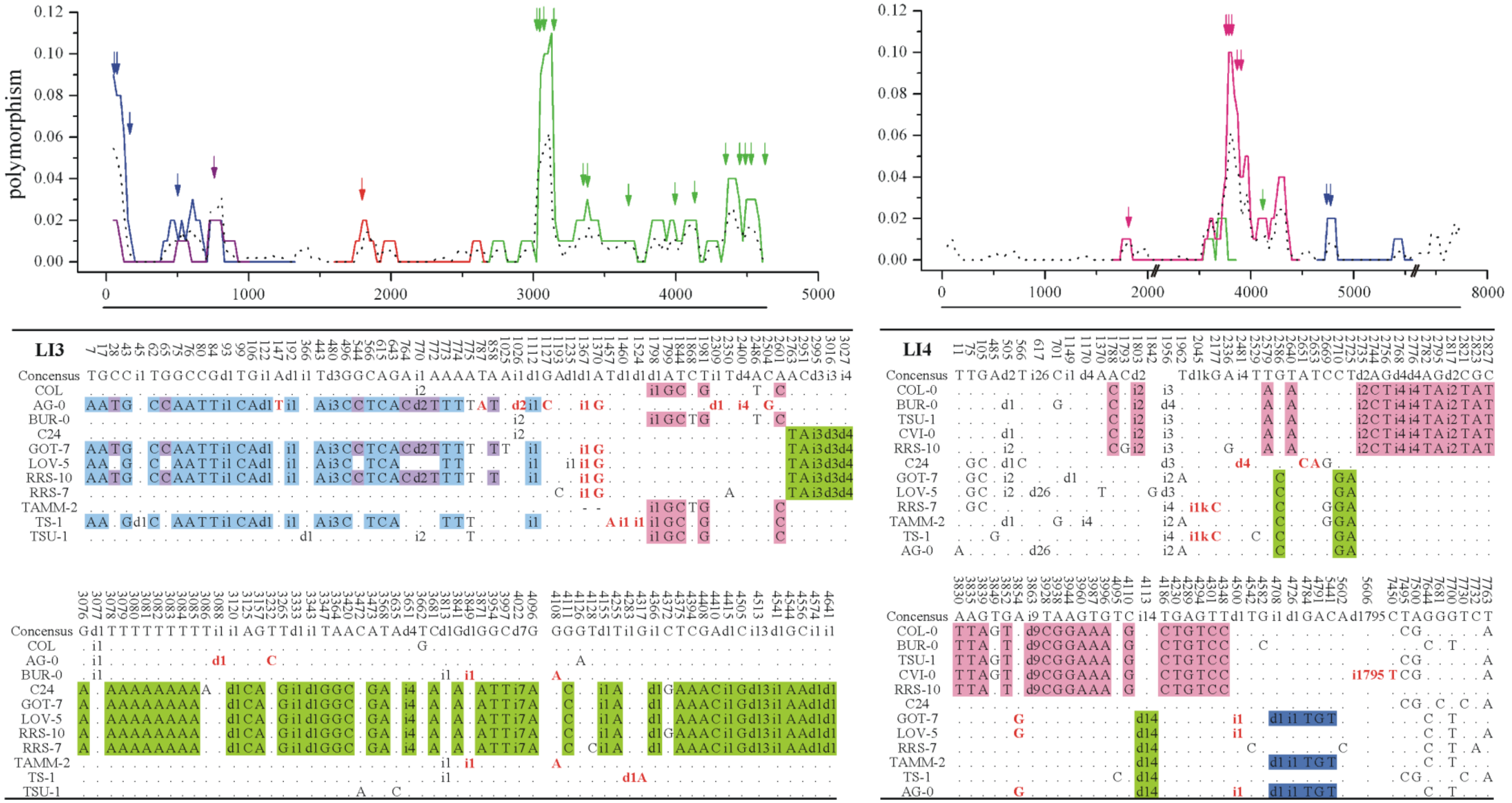

Supplementary Figure S7. Polymorphism in long intergenic locus LI2–LI4 (see LI3–4 below, in which 4.7 and 7.8kb regions were sequenced, respectively).
