# Supplementary material for: Insertions/Deletions-Associated Nucleotide Polymorphism in Arabidopsis thaliana
Source: Front Plant Sci. 2016 Nov 30;7:1792. doi: 10.3389/fpls.2016.01792 (PMC5127803; doi:10.3389/fpls.2016.01792)
Supplement: Supplementary file 13 [file Image8.PDF]

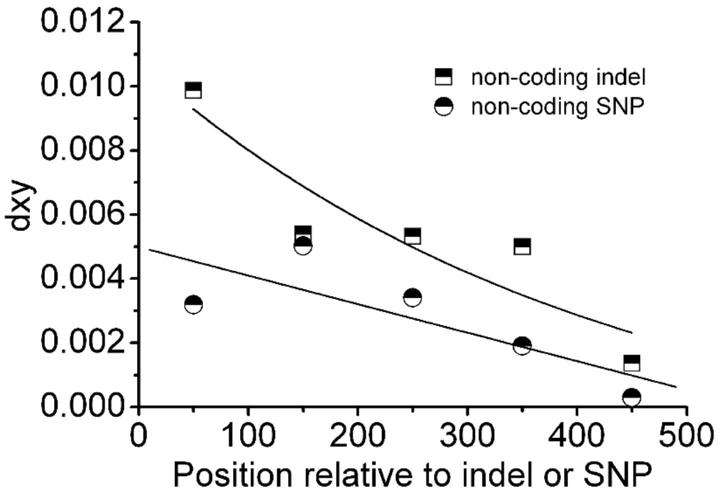

Supplementary Figure S8. Dxy distribution around non-coding indel/SNP from Nordborg data. Relationship between distance from the first indel or first SNP at a non-coding locus and the corresponding d_{xy} in the Nordborg data (the same as C but only 44 loci containing non-coding sequences). Each data point indicates the mean value of fixed nucleotide substitutions or d_{xy} in a given 100bp window.
